# Association between Fatty Acid Composition in Hair and Energy Availability during Early Lactation in Simmental and German Holstein Cows

**DOI:** 10.3390/metabo12121201

**Published:** 2022-12-01

**Authors:** Ramona Wulf, Danny Arends, Dirk Dannenberger, Thomas Ettle, Ulrich Meyer, Uwe Mohr, Gudrun A. Brockmann

**Affiliations:** 1 Albrecht Daniel Thaer-Institute, Humboldt-Universität zu Berlin, Unter den Linden 6, 10099 Berlin, Germany; 2 Department of Applied Sciences, Northumbria University, Ellison PI, Newcastle upon Tyne NE1 8ST, UK; 3 Institute of Muscle Biology and Growth, Research Institute for Farm Animal Biology (FBN), Wilhelm-Stahl-Allee 2, 18196 Dummerstorf, Germany; 4 Institute for Animal Nutrition and Feed Management, Bavarian State Research Center for Agriculture, Prof-Dürrwaechter-Platz 3, 85586 Poing, Germany; 5 Institute of Animal Nutrition, Friedrich-Loeffler-Institut, Bundesallee 37, 38116 Braunschweig, Germany; 6 Center for Agricultural Learning, Markgrafenstraße 1, 91746 Weidenbach, Germany

**Keywords:** de novo fatty acids, non-invasive indicator, lauric acid, energy balance, rumen fermentation, optiKuh

## Abstract

This study examined (1) if fatty acids in bovine hair are influenced by dietary energy levels and (2) if the relationship between energy availability and fatty acids in hair persists across breeds and farms. Sixty-two and 59 Fleckvieh (Simmental), and 55 German Holstein cows from three farms, respectively, were fed two levels of energy concentration of roughage (6.1 and 6.5 MJ net energy for lactation/kg dry matter) and two levels of concentrate supply (150 and 250 g/kg energy-corrected milk). The average body weight was 727 kg (Simmental) and 668 kg (Holstein). The average lactation number was 3.1. Hair samples were taken in lactation weeks 4 and 8. In Simmental cows, a lower energy deficit due to a relatively higher energy intake from high energy concentration of the roughage was associated with higher C18:2*n*-6 and C18:3*n*-3 contents in hair at week 8. In cows from all three farms, higher energy intake between lactation weeks 2 and 6 correlated with higher content of C18:2*n*-6 in hair samples taken in lactation weeks 4 and 8. No correlation was found for C12:0. These results provide the first evidence that increased energy intake increases the contents of C18:2*n*-6 in hair.

## 1. Introduction

Sufficient energy availability in early lactation is the basis for healthy, reproductive, and productive cows. To assess the energy balance of cows, it is necessary to determine the feed intake as well as the energy requirements for maintenance and milk production. Feed intake of cows can directly be measured using individual feeding systems, which are available on experimental farms. To evaluate the energy balance on production farms, indirect indicators are used, such as the change in body weight and body condition score [1], the fat-to-protein ratio of milk [2], fatty acids in milk [3], as well as non-esterified fatty acids and beta-hydroxybutyrate in the blood [4]. These indicators allow the identification of cows with energy deficits that cannot sufficiently adapt to the metabolic requirements of early lactation. Frequent and objective measurements are required to compensate for the influence of gut fill on body weight measurements and to assess body condition [5]. Moreover, metabolites in milk and blood are influenced by diurnal variation [6], lactation stage [7], as well as by sampling. For large populations, a potential biomarker reflecting a long time window is desired.

The fatty acid profile of the hair of cows is a potential biomarker for assessing the energy availability of early lactating cows [8,9]. Hair is a material that is non-invasively obtained and easy to store and transport. It reflects the metabolism of an individual retrospectively with a delay of about two to three weeks. Bovine hair consists of 0.2–3.1% lipids [8,9,10]. Hair lipids adhere to the hair cuticle, the cell membrane complex of the cuticle and/or the cortex. Hair lipids consist of free fatty acids, triglycerides, cholesterol esters, sterols, ceramides, wax esters, or squalene [11,12]. Hair lipids contain different fatty acids that can originate from de novo synthesis (C12:0, C14:0, part of C16:0), rumen biohydrogenation (*iso*C14:0, parts of C18:0 and C18:1*cis*-9), fat metabolism (parts of C16:0, C18:0, and C18:1*cis*-9), or feed intake (C18:2*n*-6, C18:3*n*-3). In two pilot studies, we showed that the C12:0 content in hair at lactation week 8 was higher in cows having a short interval from calving to conception and a high milk protein yield [9], as well as a high energy utilization in lactation weeks 1 to 6 [8].

Until now, it is unknown if the composition of the diet fed during early lactation influences the fatty acid profile in the hair of these cows. Therefore, the current study investigates (1) if the level of energy concentration of the roughage and the level of concentrate supply influence the fatty acid composition of hair, and (2) if the relationships between parameters of energy availability and fatty acids in the hair persist across farms, breeds, and time points of hair sampling. Based on our previous results, we tested which changes in fatty acid composition of hair are responses to diet. In particular, we expected that early lactating cows fed a higher energy level diet to have more energy available for the metabolism and, therefore, the content of the de novo synthesized fatty acid C12:0 in the hair to be higher.

## 2. Material and Methods

### 2.1. Animals

This study was performed with 62 and 59 Fleckvieh (dairy-type Simmental (SIM)) and 55 Holstein (HOL) cows from three experimental farms (farm 1 (SIM), farm 2 (SIM), farm 3 (HOL), Table 1). The dairy-type Simmental cattle are named Fleckvieh in Germany. All cows were part of the ‘optiKuh’ project (Germany), and the responsible local authority approved the animal experiments. Project-related results from farm-specific feeding trials as well as the lactation-dependent relationship between dry matter intake and diseases in different farms and breeds have already been published [13,14,15,16]. Feeding regimes were harmonized across all farms and individual feed intake was measured daily. All cows were housed in free-stall barns and had free access to feed and water *ad libitum*. On farms 2 (SIM) and 3 (HOL), cows were milked twice daily. On farm 1 (SIM), cows were milked using a milking robot with a break of at least 7 h between two consecutive milkings. The lactation number varied from 1 to 9 and was on average 3.0 ± 1.8, 3.2 ± 1.6, and 3.0 ± 1.2 (mean ± standard deviation) for farms 1 (SIM), 2 (SIM), and 3 (HOL). In farm 3 (HOL) only multiparous cows were housed.

Cows were fed a farm-specific diet before calving. After calving, cows were assigned to one of four feeding groups: (1) moderate energy concentration of roughage (6.1 MJ net energy for lactation (NE_L_)/kg dry matter (DM)) and moderate amount of concentrates (150 g/kg energy-corrected for milk (ECM)), (2) moderate energy concentration of roughage (6.1 MJ NE_L_/kg DM) and high amount of concentrates (250 g/kg ECM), (3) high energy concentration of roughage (6.5 MJ NE_L_/kg DM) and moderate amount of concentrates (150 g/kg ECM), and (4) high energy concentration of roughage (6.5 MJ NE_L_/kg DM) and high amount of concentrates (250 g/kg ECM). The lowest and highest energy contents in the diets were in feeding groups 1 and 4, respectively (Supplementary Table S1). The lower energy concentration of the roughage in feeding groups 1 and 2 was obtained by adding wheat straw. Feeding groups 3 and 4 were used on all three farms, feeding groups 1 and 2 were fed on two farms only (farms 1 and 3). The diets were provided as partial mixed rations in farms 1 and 2 (both SIM) and total mixed rations in farms 3 (HOL). The fatty acid composition in the diet was not determined. The body condition score (BCS) was determined monthly in farms 1 and 2 (both SIM), and weekly in farm 3 (HOL). At the beginning of the experiment, in lactation week 2, cows from farms 1 (SIM), 2 (SIM), and 3 (HOL) had dry matter intakes of 16 ± 3.3 kg, 17 ± 2.4 kg, and 16 ± 2.9 kg (mean ± standard deviation); milk yields of 28 ± 6.7 kg, 32 ± 5.2 kg, and 33 ± 5.1 kg; body weights of 731 ± 69 kg, 725 ± 64 kg, and 668 ± 70 kg; and BCS of 3.8 ± 0.5, 3.5 ± 0.3 kg, and 2.8 ± 0.4; respectively.

### 2.2. Hair Sampling

From each cow, one hair sample was taken from the left ventral side of the foreleg in lactation week 8 (52 ± 1.9 days in milk (DIM)) between December 2015 and March 2017. From a subset of 117 cows, an additional hair sample was obtained in lactation week 4 (28 ± 1.3 DIM). To sample native hair, consecutive hair samples were taken from neighboring skin areas. At shaving, hair samples were directly filled in zip-seal bags and then stored at −20 °C until fatty acid analysis.

### 2.3. Analysis of the Fatty Acid Composition of Bovine Hair

The analysis of the composition of fatty acids in bovine hair comprises five steps: cleaning, grinding, fat extraction, methylation of fatty acids, and determination of fatty acid methyl esters. Hair lipids were extracted from 200 mg of cleaned and mill-ground hair. The protocol was recently described in detail and is provided in Möller et al. [17]. A reference standard mixture was used to identify the different fatty acids via high-resolution gas chromatography (see supplements for a total list of fatty acids). Each sample was analyzed twice. Only fatty acids that could be reliably detected in more than 90% of animals were used for statistical analyses. The limit of quantification was 0.34 µg of specific fatty acid in 100 mg of hair. In total, 22 fatty acids were reliably detected across all farms with repeatability between 0.92 and 1.0. The repeatability σ^2^ of individual fatty acids was estimated using the variance σ^2^ of the component in the sample and the error [σ^2^ = σ^2^ sample/(σ^2^ sample + σ^2^ error)]. C8:0 could only be reliably detected in 2 out of 3 farms (farms 2 and 3). Therefore, C8:0 was not included in the downstream analysis. For polyunsaturated fatty acids, only the essential fatty acids C18:2*n*-6 and C18:3*n*-3 were above the limit of quantification. After calculating the average of the duplicates, the fatty acid composition was transformed into relative abundance for each fatty acid by setting the amount of the 22 reliably detected fatty acids to 100% (see Supplementary Table S2 for descriptive statistic of fatty acid composition of hair samples taken in weeks 4 and 8).

### 2.4. Calculation of Energy Intake, Requirements, and Balance

The phenotypic data for parameters of energy availability such as energy intake, milk yield and composition, body weight, and energy balance of lactation in weeks 2 to 6 were provided as weekly means per calendar week by the ‘optiKuh’ project [15]. To compare breeds, the energy intake was normalized per metabolic body weight. Energy requirements for maintenance and milk production were calculated according to national standards [18]:

Energy requirement for maintenance, MJ NE_L_/day = 0.293 × body weight^0.75^ [kg].

Energy requirement for milk production, MJ NE_L_/day = (0.38 × fat content [%] + 0.21 × protein content [%] + 0.95) + 0.086) × milk yield [kg/day].

Energy balance was calculated as the difference between energy intake and energy requirement for maintenance and milk production. Additionally, 15% of the body weight at calving was assumed as an energy requirement for growth for cows in the first lactation. Further energy demands were not included in the computation since all cows were not pregnant. To calculate the weekly change in body weight and BCS, a linear relationship was assumed between consecutive measurements.

### 2.5. Statistical Analysis

All analyses were performed farm-specific since all fatty acids in hair showed an influence of farm (*p* < 0.001). To convert the mean values of the calendar week to lactation weeks, the average days in milk of the calendar week were assigned to the lactation weeks 2 through 6 (week 2: 8–14 DIM; week 3: 15–21 DIM; week 4: 22–28 DIM; week 5: 29–35 DIM; week 6: 36–42 DIM). The means of energy intake and energy balance in weeks 2, 3, and 4 were influenced by the individual days in milk (*p* < 0.05). Therefore, these parameters were corrected for days in milk. Outliers were identified with more than two standard deviations less or greater than the mean and removed from the downstream data analysis. The significance threshold was *p* ≤ 0.05. Tendencies were defined with 0.05 < *p* ≤ 0.10. SAS 9.4 (SAS Institute Inc., Cary, NC, USA) and R 4.0.2 [19] was used for data analyses. Figures were prepared with Microsoft Excel (version 2016). All data are presented as least square means ± standard error unless otherwise stated.

### 2.6. Effect of Feeding Group on Parameters of Energy Availability and Fatty Acids in Hair

To investigate whether the hair fatty acid composition of lactation week 8 could be used to discriminate between the energetic level of the feeding groups, the following linear model was applied:

Y_ijk_ = µ + R_i_ + C_j_ + Lnrcl_k_ + (R × C)_ij_ + ε_ijk_

where the fixed effects are energy concentration of the roughage R_i_ (i = 6.1 or 6.5 MJ NE_L_/kg DM), amount of concentrates C_j_ (j = 150 or 250 g/kg ECM), lactation number Lnrcl_k_ (k = 1, 2, 3, ≥4), the interaction between energy concentration of the roughage and amount of concentrates (R × C)_ij_, and the residual error ε_ijk_. To examine the effects of feeding group, lactation week and lactation number on the phenotypes measured across early lactation using the abovementioned model was extended by including lactation week T_l_ (l = 2, 3, 4, 5, 6) into the model as well as the interaction between energy concentration of the roughage, concentrate supply, and week of lactation (R × C × T)_ijl_ [13]. Furthermore, the lactation week was used as a repeated measurement per cow. If the effect was significant, a mean comparison test and a correction for multiple testing using Tukey–Kramer were performed.

### 2.7. Relationship between Fatty Acids in Hair and Parameters of Energy Availability

A farm-specific correlation analysis was performed to test whether individual fatty acids in hair of lactation weeks 4 and 8 show a relationship to parameters of energy availability, independently of the feeding trial on the different farms. For this purpose, the parameters of energy availability were corrected for the feeding group and lactation number using a linear model since these effects significantly influenced the parameters of energy availability (*p* < 0.05). Since hair mirrors the metabolism retrospectively, Pearson’s correlation between the hair fatty acid profile of week 8 and parameters of energy availability in lactation weeks 2 to 6 were analyzed. Due to the limited data available around calving, the fatty acid profile of hair in week 4 was correlated only to parameters of energy availability in week 2. In the results section, only correlations that were significant to at least two farms and time points of hair samplings are reported.

## 3. Results

### 3.1. Effect of Feeding Group on Parameters of Energy Availability and Fatty Acids in Hair

#### 3.1.1. Energy Concentration of the Roughage

**Figure 1:**
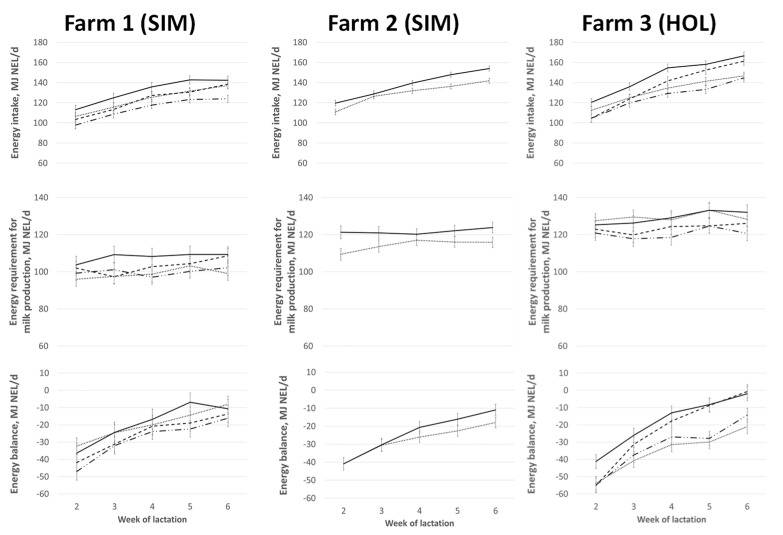
Development of energy intake, the energy requirement for milk production and maintenance, and energy balance during weeks 2 and 6 of lactation in different feeding groups; feeding groups: (1) **—** ∙ ∙ **—** moderate energy concentration of roughage (6.1 MJ NE_L_/kg DM) and a moderate amount of concentrates (150 g/kg ECM), (2) **— — —** moderate energy concentration of roughage (6.1 MJ NE_L_/kg DM) and high amount of concentrates (250 g/kg ECM), (3) ∙∙∙∙ high energy concentration of roughage (6.5 MJ NE_L_/kg DM) and a moderate amount of concentrates (150 g/kg ECM), and (4) **—** high energy concentration of roughage (6.5 MJ NE_L_/kg DM) and high amount of concentrates (250 g/kg ECM).

The effects of different concentrations of energy in the roughage could be tested in farms 1 (SIM) and 3 (HOL), where two levels were fed. In farm 2 (SIM), only one concentration was fed. Cows fed a 1.1 times higher energy concentration of the roughage also had 1.1 times higher energy intake [MJ NE_L_/d; MJ NE_L_/BW^0.75^/d] compared to cows fed a moderate energy concentration of the roughage (*p* < 0.01, Figure 1, Table 2). In both farms, the change in BCS was not influenced by the energy concentration of the roughage.

**Figure 2:**
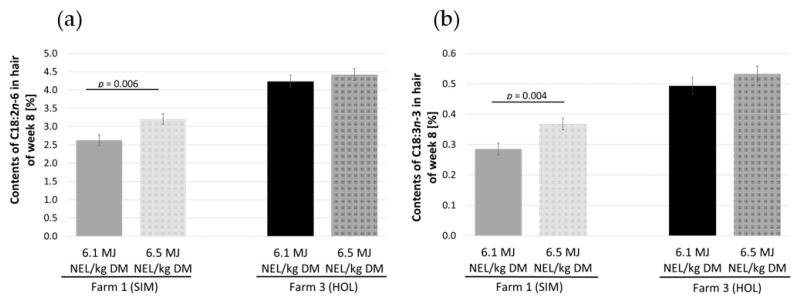
Contents of (**a**) C18:2*n*-6 and (**b**) C18:3*n*-3 in the hair of lactation week 8 based on moderate (6.1 MJ NE_L_/kg DM) and high (6.5 MJ NE_L_/kg DM) energy concentration of the roughage in farms 1 (SIM) and 3 (HOL). In farm 2 (SIM), the energy concentration of the roughage was always the same (6.5 MJ NE_L_/kg DM).

In farm 1 (SIM), the energy required for milk production in this dual-purpose breed was unaffected by energy concentration of the roughage. As a result, the energy deficit was lower in cows fed roughage with high energy concentration compared to cows fed roughage with low energy concentration (−27 ± 1.6 MJ NE_L_ vs. −19 ± 1.7 MJ NE_L_; *p* = 0.001). The observed lower negative energy deficit due to a relatively higher energy intake between weeks 2 and 6 of lactation was associated with significantly higher contents of C18:2*n*-6 (3.21 ± 0.15% vs. 2.59 ± 0.15%) and C18:3*n*-3 (0.38 ± 0.02% vs. 0.29 ± 0.02%) in the hair two weeks later in lactation week 8 (*p* < 0.01, Figure 2).

On farm 3 (HOL), the dairy cows that were fed roughage with high energy concentration used more energy for milk production. Accordingly, at this farm, the energy balance as well as the fatty acid composition in the hair were unaffected by the energy concentration of the roughage.

#### 3.1.2. Concentrate Supply

At all three farms, cows fed a high concentrate supply had higher energy intakes [MJ NE_L_/d; MJ NE_L_/BW^0.75^/d] compared to a moderate concentrate supply (*p* < 0.001, Figure 1, Table 2). In farms 1 and 2 (both SIM), the cows that were fed a high concentrate supply produced more milk and therefore used more energy for milk production. At farm 2 (SIM), the cows fed a high concentrate supply had a lower negative energy deficit in tendency than their stall mates on the moderate concentrate supply (−28 ± 1.5 MJ NE_L_ vs. −24 ± 1.5 MJ NE_L_; *p* = 0.06).

At the Holstein farm (farm 3), milk production and the energy used for milk production was unaffected by concentrate supply. Consequently, Holstein cows at farm 3 (HOL) fed a high concentrate supply had a lower energy deficit (−34 ± 1.3 MJ NE_L_ vs. −20 ± 1.3 MJ NE_L_; *p* < 0.001). At all three farms, the amount of concentrate supply did not affect the fatty acid composition in the hair (Table 3, Supplementary Table S3).

### 3.2. Interaction between Energy Concentration of the Roughage and Concentrate Supply

On farms 1 (SIM) and 3 (HOL), all four feeding groups (two levels of energy concentration of roughage and two levels of concentrate supply) were available for analysis of the interaction between energy concentration of the roughage and concentrate supply. On farm 1 (SIM), the interaction between energy concentration of the roughage and concentrate supply did not affect energy intake and energy balance. Yet, the hair fatty acid C16:0 (*p* = 0.02) was significantly influenced by the interaction of the two feed components (Table 3). However, mean comparison of the four feeding groups revealed no significant differences after correction for multiple testing.

On farm 3 (HOL), the energy requirement for maintenance (*p* < 0.001) and the energy balance (*p* = 0.04) were affected by the interaction between energy concentration of the roughage and concentrate supply. Holstein cows always showed a less pronounced energy deficit on a high than on a low concentrate supply, independently of the energy concentration of the roughage (*p* < 0.01).

### 3.3. Relationship between Fatty Acids in the Hair and Parameters of Energy Availability

Correlation analyses were performed to determine the relationship between fatty acids in the hair and parameters of energy availability across all cows per farm independent of feeding group. For this analysis, hair samples from week 8, but also from week 4 were used. No significant correlations were found between C12:0 contents of hair in weeks 4 and 8 and energy intake and energy balance in the examined period. Significant positive correlations were found between C18:2*n*-6 and C18:3*n*-3 of hair in week 8 and energy intake in weeks 2 to 6 in farm 2 (SIM) (0.36 ≤ *r* ≤ 0.41; *p* < 0.05). When the energy intake was normalized for metabolic body weight, the correlation was stronger and evident in all three farms and the two time points of hair sampling (Table 4 and Table 5). In farms 1 and 2 (both SIM), higher energy intake in week 2 normalized for the metabolic body weight significantly correlated with higher C18:2*n*-6 content in hair of lactation week 4 (0.34 ≤ *r* ≤ 0.37; *p* < 0.05). In farm 3 (HOL), a trend was found in the same direction (*r* = 0.28; *p* = 0.10). In hair taken in lactation week 8, the significant positive correlation (0.27 ≤ *r* ≤ 0.37; *p* < 0.05) between energy intake of cows in lactation weeks 4, 5, and 6 normalized for metabolic body weight and the C18:2*n*-6 content in the hair was evident in all farms independently of the breed. In addition to C18:2*n*-6, the C18:3*n*-3 content of hair in weeks 4 and 8 showed results in the same direction as C18:2*n*-6 in Simental farms 1 and 2. Higher energy intake in weeks 2, 4, 5, and 6 normalized by metabolic body weight was positively correlated with C18:3*n*-3 contents in the hair two to four weeks later (0.32 ≤ *r* ≤ 0.45; *p* < 0.05).

The relationship between energy requirement for milk production and the content of C18:2*n*-6 in the hair was diverse. In the two Simmental farms (farms 1 and 2), the relationship was in opposite directions. In farm 1 (SIM), the higher energy requirement for milk production in lactation week 5 was correlated with lower C18:2*n*-6 content in hair of week 8. In farm 2 (SIM), the relationship was in the opposite direction. In farm 3 (HOL), no correlation was found. As a consequence, in farm 1 (SIM), a lower energy deficit in week 2 and in weeks 3 to 5 was correlated with a higher content of C18:2*n*-6 in the hair of lactation weeks 4 (*r* = 0.33; *p* < 0.05) and 8 (0.27 ≤ *r* ≤ 0.45; *p* < 0.05; Supplementary Tables S4–S6).

## 4. Discussion

An optimal energy supply during early lactation is a prerequisite for robust and healthy cows. Since feed intake is not individually recorded on farms, the individual energy balance cannot be calculated. Therefore, indirect parameters, such as non-esterified fatty acids in blood [20] and fatty acid composition in milk [21] are used to assess the energy status of cows. A drawback of those metabolites is that they are influenced by the mobilization of body energy stores to compensate for the cow’s negative energy status. Another strategy is to assess the hair metabolites, which are more independent of fluxes in body fluids. Higher contents of the de novo synthesized fatty acid C12:0 in hair, for example, indicate better energy availability during periods of energy deficit in Holstein cows during early lactation [8,9]. The first studies were performed with Holstein dairy cows on a standard diet. In the current study we tested (1) if fatty acids in the hair are influenced by the energy level of the diet and (2) if the relationships between parameters of energy availability and fatty acids in hair persist across farms, breeds, and time points of hair sampling.

The amount and quality of feed taken up by a cow is the basis for an adequate energy and nutrient supply for metabolic processes such as maintenance, milk production, and reproduction. In early lactation, the insufficient feed intake cannot meet the strong demand for energy and nutrients for milk production. To minimize the energy deficit in this critical period the energy intake should be increased. Our results show that energy intake during lactation weeks 2 to 6 increases when cows are fed either a high energy concentration of the roughage or high amounts of concentrates. However, the two examined breeds, Simmental as a dual-purpose breed and Holstein as a high performing dairy breed, respond differently. While Simmental cows reached a lower energy deficit by a high energy concentration of the roughage, Holstein cows obtained a lower energy deficit by higher amounts of concentrates.

The higher uptake of diets richer in energy is associated with higher availability of nutrients for the metabolism, including, for example, the essential fatty acids C18:2*n*-6 and C18:3*n*-3. Unfortunately, we did not determine the fatty acid composition of the diets. The published data report for C18:2*n*-6 and C18:3*n*-3 showed 19–42% and 6–40% of total fatty acids in the diets, respectively [22]. Nevertheless, their content in the hair is low due to their high biohydrogenation in the rumen, the limited absorption capacity of the duodenum [23,24], their use in the metabolism for the synthesis of prostaglandins [25], and their content in milk fat [26], for example. Interestingly, the content of intermediates and products of the biohydrogenation of C18:2*n*-6 and C18:3*n*-3 in hair, such as C18:1*cis*-11 and C18:0, respectively, were not influenced by the energy concentration of the roughage. We also did not find correlations between energy intake and those produced C18:1*cis*-11 or C18:0 in hair, respectively. Consequently, we conclude that increased energy intake leads to higher amounts of C18:2*n*-6 and C18:3*n*-3 that leave the rumen unfermented and are available for storage in the hair.

This assumed higher availability of C18:2*n*-6 and C18:3*n*-3 in the blood was reflected in Simmental cows by higher contents of C18:2*n*-6 and C18:3*n*-3 in the hair of lactation week 8. In Holstein cows, this relationship was not observed. Therefore, we suggest for Holstein cows that the additionally uptaken amounts of C18:2*n*-6 and C18:3*n*-3 were fermented in the rumen, metabolized, or directly transferred to milk.

The identified correlation between low energy deficit and high C18:2*n*-6 in the hair of farm 1 (SIM), as well as between high energy intake and high C18:2*n*-6 and C18:3*n*-3 is not surprising. In a study on humans, the content of C18:2*n*-6 in blood serum was also linked to energy intake [27]. Even if the correlation coefficients identified in our study were moderate (0.27 < *r* < 0.39), they were consistent across all three farms, both breeds, and the two time points of hair sampling. In the dairy cattle breed Holstein, where increased energy intake leads to higher milk production, the relationship between energy intake and C18:2*n*-6 and C18:3*n*-3 contents in the hair was weaker than in the dual-purpose Simmental breed. It is known that milk production in the mammary gland is prioritized for energy and nutrient supply at the beginning of lactation. Depending on how much milk production is prioritized, different amounts of ingested nutrients, such as C18:2*n*-6 and C18:3*n*-3, are available for other metabolic processes and storage, including storage in the hair.

During early lactation, cows with a high energy intake and a low energy deficit are metabolically healthier [16]. For the improvement of the health of lactating cows, an accurate individual assessment of the feed intake, especially during early lactation, would be desirable. The observed positive correlation between energy intake and essential fatty acids in the hair indicate the usefulness of measuring hair fatty acids for estimating energy intake during early lactation.

Previous results demonstrated an association between high C12:0 contents in hair and a better energy availability during early lactation under a standard diet [8,9]. Recently, another study has shown that healthy Holstein cows with low beta-hydroxybutyrate levels (BHB < 0.5 mmol/L) had significantly higher amounts of C12:0 in cholesterol esters of plasma compared to sick cows with high beta-hydroxybutyrate level (BHB > 1.5 mmol/L) in early lactation [28]. We did not see such association in the current study, since the energy difference between the diet groups were only moderate. To induce diet-based differences in C12:0 contents in the hair of early lactating cows, the energy content of the diets should be more extreme or the mobilization of bodily fat depots has to be considered in addition to energy intake [8]. Even if the energy balance was in part positively influenced by the diet, previous publications from the joined optiKuh project showed that blood variables such as non-esterified fatty acids, beta-hydroxybutyrate, and adiponectin did not differ between feeding groups [13,14]. The differences in dietary energy content between the feeding groups were apparently not high enough to affect the concentration of blood parameters. Since we examined the same animals, it is not surprising that we also did not see differences between feeding groups in the C12:0 content in their hair during early lactation.

## 5. Conclusions

This is the first study to examine the relationship between parameters of energy intake, energy availability and fatty acids in the hair of two breeds, Simmental and Holstein. This study provides evidence for higher contents of C18:2*n*-6 and C18:3*n*-3 in the hair of Simmental cows fed a high energy concentration of the roughage than Simmental cows fed a low energy concentration of the roughage. In the dairy breed Holstein, we suggest that additionally uptaken amounts of C18:2*n*-6 and C18:3*n*-3 were fermented in the rumen, metabolized, or directly transferred to milk. Therefore, the relationship seen in Simmental cows was not observed.

In this study, we identified C18:2*n*-6 in hair as a marker for energy intake since the relative amount of C18:2*n*-6 significantly (*p* < 0.05) correlates with energy intake on all three farms and in the two examined breeds (Simmental and Holstein) at two time points of hair sampling (lactation weeks 4 and 8). At the two Simmental farms, in addition to C18:2*n*-6, C18:3*n*-3 was also found to correlate with energy intake. This finding provides evidence that feed intake capacity during early lactation affects the metabolism. Higher energy intake leads to higher contents of essential fatty acids in hair 2 to 4 weeks later. Therefore, the two hair fatty acids C18:2*n*-6 and C18:3*n*-3 could potentially serve as biomarkers for energy intake. The procedure of hair sampling is simple and applicable on production farms. Hence, the fatty acid composition of hair could also be applied to late- or non-lactating cattle as well as to farms where diet information as well as measurements of body weight and milk yield are not available. In particular, the hair fatty acids C18:2*n*-6 and C18:3*n*-3 should be further investigated as potential biomarkers to assess energy intake for improved animal nutrition and breeding for better energy efficiency.

With respect to the hair’s C12:0 fatty acid, a fatty acid that is mainly newly synthesized, no effects were found. Even if the energy deficit in Simmental and Holstein cows was reduced under a high energy concentration of roughage and high concentrate supply, respectively, no effect was found on the content of C12:0 in their hair. This means that C12:0 is not suitable for distinguishing between energetically well- and very well-supplied cows in early lactation. The energy difference between diets of the feeding groups has to be extreme to induce diet-based differences in C12:0 contents in the hair of early lactating cows.

## Figures and Tables

**Table 1:** Experimental setup: farms with breed, number of animals, assignments to feeding groups, lactation number, and number of hair samples in weeks 4 and 8.

			Feeding Groups (MJ NE_L_/kg DM/g/kg ECM) ^2^				Lactation Number				Hair Samples		DMI ^3^	MY ^3^	BW ^3^	BCS ^3^
Farm	Breed ^1^	*n*	6.1/150	6.1/250	6.5/150	6.5/250	1	2	3	≥4	Week 4	Week 8	kg	kg	kg	
1	SIM	62	15	15	17	15	18	9	14	21	44	62	16 ± 3.3	28 ± 6.7	731 ± 69	3.8 ± 0.5
2	SIM	59			30	29	13	8	12	26	37	59	17 ± 2.4	32 ± 5.2	725 ± 64	3.5 ± 0.3
3	HOL	55	14	13	14	14		20	22	13	36	55	16 ± 2.9	33 ± 5.1	668 ± 70	2.8 ± 0.4

^1^ SIM—Simmental; HOL—Holstein. ^2^ Values provide the energy concentration of roughage: 6.1 MJ net energy lactation (NE_L_)/kg dry matter (DM) or 6.5 MJ NE_L_/kg DM, and the amount of concentrate supply: 150 g/kg energy-corrected milk (ECM) or 250 g/kg ECM, respectively. ^3^ At the beginning of the experiment in lactation week 2; DMI—Dry matter intake; MY—Milk yield; BW—Body weight; BCS—Body condition score.

**Table 2:** Feeding group-specific effects within each farm on parameters of energy availability during lactation weeks 2 and 6. In the upper part, LS-means of the parameters per feeding group are shown; in the lower part, *p*-values are provided for the effects of the energy concentration of the roughage (R), the concentrate supply (C), the lactation number (Lactation), and the interaction between the energy concentration of the roughage and the amount of concentrate supply (R × C if any interaction is significant).

	Farm 1 (SIM)					Farm 2 (SIM)			Farm 3 (HOL)				
	Feeding Group ^1^					Feeding Group ^1^			Feeding Group ^1^				
**Parameters during Lactation Weeks 2 and 6**	**6.1/150**	**6.1/250**	**6.5/150**	**6.5/250**	**SEM ^+^**	**6.5/150**	**6.5/250**	**SEM ^+^**	**6.1/150**	**6.1/250**	**6.5/150**	**6.5/250**	**SEM ^+^**
Energy intake [MJ NE_L_/d]	114.25	122.61	123.22	131.77	1.32	129.45	137.99	1.22	126.29	136.78	131.98	147.15	1.38
Energy intake per metabolic BW [MJ NE_L_/BW^0.75^]	0.82	0.88	0.88	0.93	0.01	0.94	1.01	0.01	1.00	1.06	1.02	1.12	0.01
Energy demand													
Milk [MJ NE_L_/d]	99.95	103.01	98.89	107.98	1.30	114.36	121.69	1.21	120.48	123.57	129.29	129.14	1.08
Maintenance [MJ NE_L_/d]	41.04	40.73	40.87	41.15	0.16	40.45	39.45	0.16	36.95	39.45	37.96	38.25	0.17
Energy balance [MJ NE_L_/d]	−28.30	−25.27	−19.84	−19.02	1.27	−27.61	−23.78	1.15	−32.29	−22.70	−35.38	−18.12	1.36
Delta body weight [kg]	−5.80	−4.90	−5.75	−2.09	1.09	−7.12	−7.15	0.93	−8.79	−10.61	−6.81	−8.28	0.94
Delta body condition score	−0.07	−0.11	−0.07	−0.08	0.01	−0.05	−0.05	0.00	−0.10	−0.08	−0.07	−0.08	0.01

	**Farm 1 (SIM)**					**Farm 2 (SIM)**			**Farm 3 (HOL)**				
	**R**	**C**	**Week**	**Lactation**		**C**	**Week**	**Lactation**	**R**	**C**	**Week**	**Lactation**	**R ×** **C**
Energy intake [MJ NE_L_/d]	<0.001	<0.001	<0.001	<0.001		<0.001	<0.001	<0.001	<0.001	<0.001	<0.001	0.237	0.206
Energy intake per metabolic BW [MJ NE_L_/BW^0.75^]	<0.001	<0.001	<0.001	<0.001		<0.001	<0.001	<0.001	0.006	<0.001	<0.001	0.013	0.403
Energy demand													
Milk [MJ NE_L_/d]	0.281	0.001	0.412	<0.001		<0.001	0.612	<0.001	<0.001	0.416	0.270	<0.001	0.379
Maintenance [MJ NE_L_/d]	0.623	0.952	0.648	<0.001		<0.001	0.192	<0.001	0.754	<0.001	0.665	<0.001	<0.001
Energy balance [MJ NE_L_/d]	0.001	0.409	<0.001	0.001		0.063	<0.001	0.515	0.691	<0.001	<0.001	<0.001	0.043
Delta body weight [kg]	0.495	0.293	<0.001	0.730		0.987	<0.001	0.314	0.190	0.310	<0.001	0.449	0.918
Delta BCS	0.227	0.090	0.073	0.017		0.854	<0.001	0.083	0.176	0.929	<0.001	0.970	0.257

^1^ Values in the feeding group provide the energy concentration of roughage (6.1 or 6.5 MJ NE_L_/kg DM) and the amount of concentrate supply (150 or 250 g/kg ECM). In farm 2 (SIM) only one energy concentration of the roughage was provided (6.5 MJ NEL/kg DM). ^+^ standard error of the mean, MJ—megajoule, NE_L_—net energy for lactation, BW—body weight. Non-significant *p*-values > 0.05 of interaction terms are not shown (e.g., × R × C × Week in all three farms, R × C in farm 1).

**Table 3:** Feeding group-specific effects within each farm on the fatty acid composition of hair taken in lactation week 8. In the upper part, LS-means of the relative amount of every fatty acid (% of total fatty acids) per feeding group is shown, in the lower part, *p*-values are given for the effects of the energy concentration of the roughage (R), the concentrate supply (C), the lactation number (Lactation), and the interaction between the energy concentration of the roughage and the amount of concentrate supply (R × C in farm 1 and 3).

	**Farm 1 (SIM)**					**Farm 2 (SIM)**			**Farm 3 (HOL)**				
	**Feeding Group ^1^**					**Feeding Group ^1^**			**Feeding Group ^1^**				
	**6.1/150**	**6.1/250**	**6.5/150**	**6.5/250**	**SEM ^+^**	**6.5/150**	**6.5/250**	**SEM ^+^**	**6.1/150**	**6.1/250**	**6.5/150**	**6.5/250**	**SEM ^+^**
C10:0	5.5	6.2	6.1	5.8	0.17	5.4	5.6	0.14	6.2	6.4	6.9	6.3	0.26
C12:0	4.7	4.9	4.8	4.4	0.09	4.4	4.4	0.08	4.0	4.1	4.0	4.1	0.08
C14:0	30.4	31.6	30.7	29.6	0.68	28.7	29.3	0.52	23.4	24.4	24.8	23.6	0.53
C16:0	22.9	21.9	21.7	22.9	0.23	20.8	20.0	0.26	22.3	22.4	22.2	22.6	0.25
C18:0	12.7	12.3	12.5	12.6	0.26	12.8	12.6	0.20	15.3	15.4	15.5	15.4	0.29
C18:1*cis*-9	6.1	5.7	6.3	6.9	0.23	6.4	7.1	0.34	7.0	7.6	6.5	7.1	0.20
C18:2*n*-6	2.7	2.6	3.3	3.1	0.11	4.5	4.3	0.16	4.3	4.2	4.3	4.6	0.11
C18:3*n*-3	0.3	0.3	0.4	0.4	0.01	0.5	0.5	0.03	0.5	0.5	0.5	0.5	0.02

	**Farm 1 (SIM)**					**Farm 2 (SIM)**			**Farm 3 (HOL)**				
	***p*** **-values**					***p*** **-values**			***p*** **-values**				
	**R**	**C**	**Lactation**		**R × C**	**C**	**Lactation**		**R**	**C**	**Lactation**		**R × C**
C10:0	0.686	0.564	0.009		0.142	0.486	0.295		0.547	0.745	0.266		0.431
C12:0	0.388	0.634	0.254		0.074	0.687	0.166		0.891	0.456	0.017		0.789
C14:0	0.53	0.996	0.067		0.392	0.507	0.066		0.753	0.912	0.046		0.336
C16:0	0.776	0.906	0.303		0.022	0.143	0.444		0.938	0.616	0.190		0.75
C18:0	0.934	0.733	0.136		0.628	0.682	0.146		0.885	0.973	0.119		0.814
C18:1*cis*-9	0.131	0.785	0.028		0.266	0.291	0.27		0.265	0.163	0.269		0.976
C18:2*n*-6	0.006	0.532	0.161		0.698	0.722	0.681		0.426	0.758	0.506		0.383
C18:3*n*-3	0.004	0.565	0.037		0.710	0.418	0.967		0.294	0.382	0.526		0.967

^1^ Values in the feeding group provide the energy concentration of roughage (6.1 or 6.5 MJ NE_L_/kg DM) and the amount of concentrate supply (150 or 250 g/kg ECM). In farm 2 (SIM) only one energy concentration of the roughage was provided (6.5 MJ NE_L_/kg DM), ^+^ standard error of the mean.

**Table 4:** Pearson’s correlation coefficients between energy intake [MJ NE_L_/d] in lactation weeks 2 to 6 corrected for feeding group and lactation number in farm 1 (SIM), farm 2 (SIM), and farm 3 (HOL), and C18:2*n*-6 and C18:3*n*-3 contents in the hair of weeks 4 and 8.

		Energy Intake [MJ NE_L_/d] in Lactation Weeks					
		2	2	3	4	5	6
C18:2*n*-6 in hair		of week 4	of week 8				
Farm 1 (SIM)		0.27 ^+^	−0.10	0.08	0.27 ^+^	0.19	0.25 ^+^
Farm 2 (SIM)		0.23	0.27 ^+^	0.36 *	0.28 *	0.37 **	0.41 **
Farm 3 (HOL)		0.15	−0.10	−0.11	0.12	0.08	0.15
C18:3*n*-3 in hair		of week 4	of week 8				
Farm 1 (SIM)		0.26 ^+^	−0.07	0.01	0.18	0.23 ^+^	0.24 ^+^
Farm 2 (SIM)		0.26	0.46 **	0.43 **	0.39 **	0.45 **	0.47 ***
Farm 3 (HOL)		0.12	−0.08	−0.15	0.03	0.03	0.12

^+^ (*p* ≤ 0.1), * (*p* ≤ 0.05), ** (*p* ≤ 0.01), and *** (*p* ≤ 0.001) refer to suggestive, significant, and highly significant correlations.

**Table 5:** Pearson’s correlation coefficients between energy intake per metabolic body weight (MJ NE_L_/BW^0.75^/d) in lactation weeks 2 to 6 corrected for feeding group and lactation number in farm 1 (SIM), farm 2 (SIM), and farm 3 (HOL), and C18:2*n*-6 and C18:3*n*-3 contents in the hair of weeks 4 and 8.

	Energy Intake per Metabolic Body Weight [MJ NE_L_/BW^0.75^/d] in Lactation Weeks					
	2	2	3	4	5	6
C18:2*n*-6 in hair	of week 4	of week 8				
Farm 1 (SIM)	0.37 *	0.03	0.21	0.37 **	0.26 *	0.18
Farm 2 (SIM)	0.34 *	0.29^+^	0.39 **	0.33 *	0.31 *	0.33 **
Farm 3 (HOL)	0.28 ^+^	0.05	0.08	0.27 ^+^	0.21	0.27 *
C18:3*n*-3 in hair	of week 4	of week 8				
Farm 1 (SIM)	0.39 *	0.07	0.14	0.25 ^+^	0.32 **	0.20
Farm 2 (SIM)	0.33 *	0.45 **	0.45 **	0.39 **	0.38 **	0.35 **
Farm 3 (HOL)	0.21	−0.01	0.01	0.15	0.09	0.18

^+^ (*p* ≤ 0.1), * (*p* ≤ 0.05), and ** (*p* ≤ 0.01) refer to suggestive, significant, and highly significant correlations.

## Data Availability

The data presented in this study are available on reasonable request from the corresponding author.

## Supplementary Materials

The following supporting information can be downloaded at: https://www.mdpi.com/article/10.3390/metabo12121201/s1, Table S1: Composition of diets; Table S2: Descriptive statistic of fatty acid composition (% of total fatty acids) of hair samples taken in week 4 and 8 of lactation in farm 1, 2 and 3 (mean + sd); Table S3: Farm-specific effects of energy concentration of the roughage (R), amount of concentrate supply (C), lactation week (lwo), lactation number class (Lactation) on fatty acid composition (% of total fatty acids) of hair taken in week 8 of lactation; Table S4: Pearson correlation coefficients (r) and *p*-values (in brackets) between fatty acids (% of total fatty acids) of hair in week 4 and 8 and parameters of energy availability in week 2 to 6 on farm 1 (SIM); Table S5: Pearson correlation coefficients (r) and *p*-values (in brackets) between fatty acids (% of total fatty acids) of hair in week 4 and 8 and parameters of energy availability in week 2 to 6 on farm 2 (SIM); Table S6: Pearson correlation coefficients (r) and *p*-values (in brackets) between fatty acids (% of total fatty acids) of hair in week 4 and 8 and parameters of energy availability in week 2 to 6 on farm 3 (HOL)
