# Supplementary material for: Association between Fatty Acid Composition in Hair and Energy Availability during Early Lactation in Simmental and German Holstein Cows
Source: Metabolites. 2022 Dec 1;12(12):1201. doi: 10.3390/metabo12121201 (PMC9781642; doi:10.3390/metabo12121201)
Supplement: Supplementary file 1 [file metabolites-12-01201-s001.zip › metabolites-1985875-supplementary.pdf]

Supplementary material

Suppl. Table S1: Composition of diets

	Farm 1 (SIM)				Farm 2 (SIM)		Farm 3 (HOL)			
Feeding groups	6.1/150	6.1/250	6.5/150	6.5/250	6.5/150	6.5/250	6.1/150	6.1/250	6.5/150	6.5/250
Diet provided as	PMR				PMR		TMR			
Dry matter [g/kg]	476	514	450	476	466	514	547	601	523	580
Chemical composition [g/kg of dry matter]										
Crude ash	64	65	64	65	71	70	66	65	67	65
Crude protein	148	159	153	158	158	159	129	138	135	142
Crude fat	36	36	37	37	45	44	37	39	38	40
Crude fibre	208	190	183	177	177	167	205	184	188	173
NDF om	417	387	377	368	377	362	404	366	372	345
ADF om	251	234	223	217	222	212	241	221	221	207
Energy [MJ/kg DM]										
NE_L	6.6	6.8	6.8	6.9	6.8	6.8	6.7	7.1	6.9	7.2
ME	10.8	11.1	11.2	11.3	11.1	11.1	11.0	11.5	11.3	11.7

Feeding groups: 6.1/150: 6.1 MJ NE_L energy concentration of the roughage/kg DM and 150 g concentrate supply /kg ECM; 6.1/250: 6.1 MJ NE_L energy concentration of the roughage/kg DM and 250 g concentrate supply /kg ECM; 6.5/150: 6.5 MJ NE_L energy concentration of the roughage/kg DM and 150 g concentrate supply /kg ECM; 6.5/250: 6.5 MJ NE_L energy concentration of the roughage/kg DM and 250 g concentrate supply /kg ECM;
Diets provided either as total mixed ration (TMR; roughage and concentrate mixed) or partial mixed ration (PMR; roughage and concentrate as separate components); NDF - Neutral detergent fibre; ADF - Acid detergent fibre; om - organic matter; NE_L – net energy of lactation; ME – metabolizable energy
Reference: Urh C. Denissen J. Harder I. Koch C. Gerster E. Ettle T. Kraus N. Schmitz R. Kuhla B. Stamer E. Spiekers H and Sauerwein H 2019. Circulating adiponectin concentrations during the transition from pregnancy to lactation in high-yielding dairy cows: testing the effects of farm. parity. and dietary energy level in large animal numbers. Domestic Animal Endocrinology 69. 1-12

Composition of reference standard mixture:

- Even chain saturated fatty acids: C8:0, C10:0, C12:0, C14:0, C16:0, C18:0, C20:0, C22:0, C24:0, C26:0
- Odd chain saturated fatty acids: C11:0, C13:0, C15:0, C17:0, C21:0, C23:0
- Branched chain fatty acids: *iso*C13:0, *anteiso*C13:0, *iso*C14:0, *iso*C15:0, *anteiso*C15:0, *iso*C16:0
- Monounsaturated fatty acids: C14:1, C16:1, C17:1, C18:1*trans*-9, C18:1*trans*-11, C18:1*cis*-9, C18:1*cis*-11, C20:1, C22:1, C24:1
- Polyunsaturated fatty acids: C18:2*trans*, C18:2*n*-6, C18:3*n*-6, C18:3*n*-3, CLA*cis*-9, *trans*-11, C20:2*n*-6, C20:3*n*-6, C20:3*n*-3, C20:4*n*-6, C22:2*n*-6, C22:4*n*-6, C22:6*n*-3

Suppl. Table S2: Descriptive statistic of fatty acid composition (% of total fatty acids) of hair samples taken in weeks 4 and 8 of lactation in farm 1, 2 and 3 (mean +sd)

	Farm 1 (SIM)		Farm 2 (SIM)		Farm 3 (HOL)	
Hair sample	week 4	week 8	week 4	week 8	week 4	week 8
n	44	62	37	59	36	55
C10:0	5.85±0.93	6.06±1.3	5.25±1.01	5.45±1.02	9.02±4.44	6.41±1.91
C12:0	4.48±0.59	4.77±0.69	3.96±0.67	4.38±0.61	4.02±0.79	4.08±0.59
C14:0	31.06±4.32	31.01±5.39	28.48±5.2	28.91±3.87	26.22±5.44	24.15±3.83
C16:0	22.57±1.96	22.2±1.78	22.30±2.6	20.64±1.98	21.49±2.20	22.29±1.79
C18:0	12.29±1.66	12.37±1.98	12.84±1.82	12.75±1.47	13.29±1.69	15.37±2.14
C20:0	2.80±0.49	2.78±0.62	3.66±0.65	3.26±0.57	2.38±0.29	2.33±0.29
C22:0	1.47±0.19	1.50±0.21	1.64±0.20	1.64±0.26	1.95±0.27	1.88±0.19
C24:0	2.34±0.30	2.24±0.32	2.43±0.46	2.62±0.44	2.63±0.50	2.55±0.35
C26:0	0.54±0.10	0.52±0.09	0.83±0.19	0.85±0.18	0.49±0.11	0.51±0.09
C13:0	0.28±0.05	0.26±0.04	0.22±0.04	0.23±0.04	0.30±0.07	0.31±0.05
C15:0	0.76±0.09	0.74±0.09	0.70±0.13	0.70±0.11	0.89±0.14	0.98±0.13
C17:0	0.75±0.11	0.74±0.13	1.10±0.17	1.06±0.13	0.68±0.10	0.75±0.10
C21:0	0.20±0.04	0.20±0.04	0.23±0.03	0.23±0.04	0.21±0.03	0.23±0.03
*iso*C14:0	0.32±0.15	0.29±0.11	0.36±0.24	0.38±0.16	0.37±0.09	0.38±0.10
*anteiso*C15:0	0.23±0.05	0.24±0.05	0.20±0.05	0.23±0.08	0.25±0.05	0.34±0.10
C16:1	0.77±0.13	0.78±0.20	0.79±0.19	0.76±0.17	0.71±0.09	0.81±0.14
C18:1*cis*-9	6.13±1.51	6.16±1.80	5.99±1.82	6.54±2.52	6.40±1.49	7.01±1.48
C18:1*cis*-11	2.77±0.67	2.67±0.71	3.04±0.76	2.75±0.57	2.50±0.68	2.75±0.56
C20:1	0.76±0.19	0.75±0.21	0.94±0.24	0.94±0.24	0.55±0.15	0.56±0.12
C22:1	0.21±0.05	0.22±0.06	0.27±0.06	0.27±0.07	0.22±0.05	0.24±0.05
C18:2*n*-6	2.55±0.82	2.91±0.84	3.34±1.01	4.32±1.20	3.69±0.56	4.36±0.81
C18:3*n*-3	0.31±0.14	0.32±0.12	0.43±0.19	0.49±0.19	0.47±0.13	0.52±0.13

Suppl. Table S3: Farm-specific effects of energy concentration of the roughage (R), amount of concentrate supply (C), lactation week (lwo), lactation number class (Lactation) on fatty acid composition (% of total fatty acids) of hair taken in week 8 of lactation

	Farm 1 (SIM)								Farm 2 (SIM)			
	Feeding group				*p*-values				Feeding group		*p*-values	
	6.1/150	6.1/250	6.5/150	6.5/250	R	C	Lactation	R×C	6.5/150	6.5/250	C	Lactation
C10:0	5.5±0.35	6.2±0.32	6.1±0.31	5.8±0.32	0.686	0.564	**0.009**	0.142	5.4±0.2	5.6±0.21	0.486	0.295
C12:0	4.7±0.18	4.9±0.18	4.8±0.17	4.4±0.19	0.388	0.634	0.254	0.074	4.4±0.12	4.4±0.13	0.687	0.166
C14:0	30.4±1.45	31.6±1.36	30.7±1.33	29.6±1.39	0.530	0.996	0.067	0.392	28.7±0.72	29.3±0.80	0.507	0.066
C16:0	22.9±0.50	21.9±0.45	21.7±0.45	22.9±0.49	0.776	0.906	0.303	**0.022**	20.8±0.38	20.0±0.41	0.143	0.444
C18:0	12.7±0.54	12.3±0.55	12.5±0.52	12.6±0.52	0.934	0.733	0.136	0.628	12.8±0.28	12.6±0.31	0.682	0.146
C20:0	2.8±0.17	2.8±0.16	2.8±0.16	2.9±0.16	0.971	0.690	0.172	0.820	3.3±0.10	3.1±0.11	0.468	**0.015**
C22:0	1.5±0.06	1.6±0.06	1.5±0.05	1.4±0.06	**0.019**	0.977	0.945	0.270	1.6±0.05	1.6±0.05	0.947	0.708
C24:0	2.3±0.09	2.3±0.09	2.2±0.08	2.2±0.09	0.070	0.998	0.734	0.869	2.6±0.09	2.6±0.09	0.611	0.531
C26:0	0.5±0.03	0.5±0.03	0.5±0.02	0.5±0.03	0.283	0.806	0.699	0.985	0.8±0.04	0.9±0.04	0.402	0.849
C13:0	0.3±0.01	0.3±0.01	0.3±0.01	0.3±0.01	0.433	0.745	0.661	0.451	0.2±0.01	0.2±0.01	0.238	0.804
C15:0	0.8±0.02	0.7±0.02	0.7±0.02	0.7±0.02	0.487	0.175	**0.016**	0.057	0.7±0.02	0.7±0.02	0.872	0.067
C17:0	0.8±0.04	0.7±0.03	0.8±0.03	0.7±0.03	0.880	0.373	**0.039**	0.593	1.1±0.03	1.0±0.03	0.339	0.393
C21:0	0.2±0.01	0.2±0.01	0.2±0.01	0.2±0.01	0.637	0.503	0.068	0.991	0.2±0.01	0.2±0.01	0.310	0.491
isoC14:0	0.2±0.01	0.2±0.01	0.2±0.01	0.2±0.01	0.486	0.727	0.088	0.368	0.2±0.01	0.2±0.01	0.697	0.353
anteisoC15:0	0.3±0.01	0.2±0.01	0.2±0.01	0.3±0.01	0.533	0.787	**0.028**	**0.042**	0.2±0.01	0.2±0.02	0.527	0.526
C16:1	0.8±0.05	0.7±0.05	0.8±0.05	0.8±0.05	0.369	0.415	0.071	0.295	0.7±0.04	0.8±0.04	0.586	0.756
C18:1cis-9	6.1±0.46	5.7±0.45	6.3±0.42	6.9±0.44	0.131	0.785	**0.028**	0.266	6.4±0.50	7.1±0.52	0.291	0.270
C18:1cis-11	2.9±0.20	2.6±0.19	2.6±0.19	2.9±0.19	0.948	0.921	0.490	0.113	2.8±0.11	2.6±0.12	0.372	0.781
C20:1	0.7±0.06	0.8±0.06	0.7±0.05	0.8±0.06	0.664	0.303	0.621	0.865	0.9±0.05	0.9±0.05	0.782	0.188
C22:1	0.2±0.02	0.2±0.02	0.2±0.01	0.2±0.02	0.894	0.848	0.317	0.889	0.3±0.01	0.3±0.01	0.992	0.558
C18:2-6	2.7±0.22	2.6±0.20	3.3±0.20	3.1±0.21	**0.006**	0.532	0.161	0.698	4.5±0.24	4.3±0.25	0.722	0.681
C18:3n-3	0.3±0.03	0.3±0.03	0.4±0.03	0.4±0.03	**0.004**	0.565	**0.037**	0.710	0.5±0.04	0.5±0.04	0.418	0.967

Continued Suppl. Table S3

	Farm 3 (HOL)							
	Feeding group				P-values			
	6.1/150	6.1/250	6.5/150	6.5/250	R	C	Lactation	R×C
C10:0	6.2±0.59	6.4±0.53	6.9±0.51	6.3±0.54	0.547	0.745	0.266	0.431
C12:0	4.0±0.17	4.1±0.16	4.0±0.16	4.1±0.15	0.891	0.456	**0.017**	0.789
C14:0	23.4±1.13	24.4±1.04	24.8±1.00	23.6±1.05	0.753	0.912	**0.046**	0.336
C16:0	22.3±0.55	22.4±0.5	22.2±0.48	22.6±0.53	0.938	0.616	0.190	0.750
C18:0	15.3±0.63	15.4±0.63	15.5±0.60	15.4±0.59	0.885	0.973	0.119	0.814
C20:0	2.4±0.09	2.3±0.08	2.4±0.08	2.3±0.08	0.856	0.417	0.150	0.844
C22:0	1.9±0.06	1.8±0.06	1.9±0.05	1.9±0.05	0.542	0.533	0.992	0.651
C24:0	2.5±0.11	2.5±0.10	2.6±0.09	2.5±0.10	0.624	0.465	0.127	0.509
C26:0	0.5±0.03	0.5±0.02	0.5±0.02	0.5±0.02	0.760	0.691	0.208	0.428
C13:0	0.3±0.02	0.3±0.02	0.3±0.01	0.3±0.02	0.965	0.978	0.145	0.596
C15:0	1.0±0.04	1.0±0.04	1.0±0.04	1.0±0.04	0.484	0.652	0.681	0.956
C17:0	0.8±0.03	0.8±0.03	0.8±0.03	0.7±0.03	0.450	0.682	0.117	0.695
C21:0	0.2±0.01	0.2±0.01	0.2±0.01	0.2±0.01	0.748	0.790	0.715	0.848
isoC14:0	0.2±0.01	0.2±0.01	0.2±0.01	0.2±0.01	0.127	0.894	0.494	0.803
anteisoC15:0	0.3±0.03	0.4±0.03	0.3±0.03	0.3±0.03	0.927	0.619	0.611	0.893
C16:1	0.9±0.04	0.8±0.04	0.8±0.03	0.8±0.04	0.406	0.903	**0.001**	0.083
C18:1cis-9	7.0±0.45	7.6±0.41	6.5±0.39	7.1±0.40	0.265	0.163	0.269	0.976
C18:1cis-11	2.8±0.17	2.9±0.16	2.6±0.14	2.8±0.15	0.295	0.370	**0.033**	0.985
C20:1	0.6±0.04	0.6±0.03	0.6±0.03	0.5±0.03	0.861	0.417	**0.040**	0.584
C22:1	0.3±0.01	0.2±0.01	0.2±0.01	0.2±0.01	0.125	0.413	0.423	0.873
C18:2-6	4.3±0.24	4.2±0.23	4.3±0.22	4.6±0.23	0.426	0.758	0.506	0.383
C18:3n-3	0.5±0.04	0.5±0.04	0.5±0.03	0.5±0.04	0.294	0.382	0.526	0.967

Feeding groups: 6.1/150: 6.1 MJ NE_L energy concentration of the roughage/kg DM and 150 g concentrate supply /kg ECM; 6.1/250: 6.1 MJ NE_L energy concentration of the roughage/kg DM and 250 g concentrate supply /kg ECM; 6.5/150: 6.5 MJ NE_L energy concentration of the roughage/kg DM and 150 g concentrate supply /kg ECM; 6.5/250: 6.5 MJ NE_L energy concentration of the roughage/kg DM and 250 g concentrate supply /kg ECM; Values presented as LS-means

Suppl. Table S4: Pearson correlation coefficients (r) and P-values (in brackets) between fatty acids (% of total fatty acids) of hair in week 4 and 8 and parameters of energy availability in week 2 to 6 on farm 1 (SIM)

	Hair sample week 4															
Lactation week 2	C10:0		C12:0		C14:0		C16:0		C18:0		C18:1*cis*-9		C18:2*n*-6		C18:3*n*-3	
Dry matter intake [kg DM]	0.20	(0.20)	0.20	(0.19)	0.18	(0.25)	-0.23	(0.14)	-0.24	(0.13)	-0.15	(0.34)	0.26	(0.09)	0.27	(0.08)
Energy intake [MJ NE_L]	0.18	(0.25)	0.19	(0.23)	0.17	(0.28)	-0.19	(0.22)	-0.25	(0.10)	-0.13	(0.40)	0.27	(0.08)	0.26	(0.10)
Dry matter intake per metabolic BW [kg DM/ $BW^{0.75}$]	0.21	(0.19)	0.20	(0.21)	0.21	(0.18)	-0.34	(0.03)	-0.23	(0.14)	-0.15	(0.33)	0.35	(0.02)	0.40	(0.01)
Energy intake per metabolic BW [MJ NE_L/ $BW^{0.75}$]	0.20	(0.21)	0.19	(0.24)	0.21	(0.18)	-0.31	(0.04)	-0.26	(0.10)	-0.14	(0.36)	0.37	(0.02)	0.39	(0.01)
Energy balance [MJ NE_L]	-0.06	(0.69)	0.00	(1.00)	-0.04	(0.79)	-0.09	(0.58)	0.04	(0.81)	-0.03	(0.83)	0.32	(0.04)	0.31	(0.05)
Maintenance [MJ NE_L]	-0.09	(0.59)	-0.06	(0.70)	-0.15	(0.34)	0.25	(0.11)	0.01	(0.95)	0.14	(0.37)	-0.09	(0.56)	-0.14	(0.38)
Milk [MJ NE_L]	0.12	(0.44)	0.16	(0.32)	0.09	(0.57)	-0.08	(0.63)	-0.08	(0.60)	-0.07	(0.64)	-0.06	(0.68)	-0.05	(0.77)
BCS	-0.06	(0.74)	0.02	(0.91)	-0.05	(0.75)	0.12	(0.48)	-0.05	(0.76)	0.15	(0.35)	0.07	(0.67)	0.01	(0.96)
Delta body weight	-0.09	(0.55)	-0.03	(0.84)	-0.05	(0.77)	0.04	(0.80)	0.00	(0.98)	0.10	(0.54)	0.01	(0.95)	0.01	(0.93)
Delta BCS	-0.04	(0.84)	0.07	(0.73)	0.01	(0.96)	0.07	(0.69)	0.06	(0.76)	-0.18	(0.34)	-0.04	(0.84)	-0.11	(0.55)

	Hair sample week 8															
Lactation week 2	C10:0		C12:0		C14:0		C16:0		C18:0		C18:1*cis*-9		C18:2*n*-6		C18:3*n*-3	
Dry matter intake [kg DM]	0.01	(0.95)	0.16	(0.25)	0.06	(0.67)	-0.03	(0.84)	0.03	(0.84)	-0.21	(0.11)	-0.09	(0.50)	-0.06	(0.68)
Energy intake [MJ NE_L]	0.02	(0.91)	0.15	(0.27)	0.06	(0.67)	-0.02	(0.90)	0.02	(0.91)	-0.20	(0.14)	-0.10	(0.47)	-0.07	(0.63)
Dry matter intake per metabolic BW [kg DM/ $BW^{0.75}$]	-0.06	(0.67)	0.09	(0.54)	-0.01	(0.97)	-0.08	(0.54)	0.06	(0.64)	-0.12	(0.39)	0.04	(0.74)	0.08	(0.54)
Energy intake per metabolic BW [MJ NE_L/ $BW^{0.75}$]	-0.05	(0.70)	0.08	(0.57)	0.00	(0.97)	-0.07	(0.60)	0.05	(0.71)	-0.10	(0.45)	0.03	(0.81)	0.07	(0.60)
Energy balance [MJ NE_L]	0.05	(0.70)	0.03	(0.84)	0.04	(0.77)	-0.13	(0.35)	-0.03	(0.80)	-0.07	(0.61)	0.08	(0.55)	0.12	(0.37)
Maintenance [MJ NE_L]	-0.02	(0.88)	0.04	(0.75)	0.04	(0.77)	0.15	(0.29)	-0.07	(0.63)	-0.05	(0.75)	-0.10	(0.48)	-0.17	(0.22)
Milk [MJ NE_L]	-0.13	(0.35)	-0.07	(0.59)	-0.07	(0.61)	0.15	(0.27)	0.13	(0.34)	0.06	(0.67)	-0.21	(0.12)	-0.15	(0.27)
BCS	0.09	(0.53)	0.14	(0.34)	0.03	(0.85)	0.05	(0.73)	0.02	(0.87)	-0.09	(0.51)	-0.31	(0.03)	-0.30	(0.04)
Delta body weight	0.10	(0.46)	0.19	(0.18)	0.15	(0.26)	-0.03	(0.83)	-0.19	(0.16)	-0.17	(0.21)	-0.05	(0.72)	-0.08	(0.58)
Delta BCS	0.08	(0.61)	-0.05	(0.77)	0.12	(0.44)	-0.02	(0.92)	-0.12	(0.46)	-0.07	(0.66)	0.05	(0.78)	-0.08	(0.64)
Lactation week 3																
Dry matter intake [kg DM]	-0.05	(0.74)	-0.07	(0.62)	0.02	(0.86)	0.10	(0.49)	0.02	(0.86)	0.03	(0.82)	0.08	(0.58)	0.00	(0.99)

Energy intake [MJ NE_L]	-0.09	(0.51)	-0.09	(0.52)	-0.03	(0.84)	0.15	(0.30)	0.06	(0.65)	0.07	(0.59)	0.08	(0.57)	0.01	(0.96)
Dry matter intake per metabolic BW [kg DM/ $BW^{0.75}$]	-0.07	(0.61)	-0.10	(0.50)	-0.01	(0.97)	0.05	(0.73)	0.04	(0.77)	0.09	(0.53)	0.22	(0.11)	0.13	(0.34)
Energy intake per metabolic BW [MJ NE_L/ $BW^{0.75}$]	-0.12	(0.37)	-0.12	(0.39)	-0.06	(0.65)	0.11	(0.45)	0.09	(0.53)	0.14	(0.33)	0.21	(0.11)	0.14	(0.31)
Energy balance [MJ NE_L]	-0.02	(0.91)	-0.14	(0.34)	-0.15	(0.29)	0.07	(0.63)	-0.05	(0.73)	0.09	(0.52)	0.27	(0.05)	0.20	(0.15)
Maintenance [MJ NE_L]	0.11	(0.43)	-0.01	(0.95)	0.08	(0.58)	0.04	(0.79)	-0.12	(0.40)	-0.08	(0.59)	-0.14	(0.32)	-0.25	(0.07)
Milk [MJ NE_L]	0.04	(0.78)	-0.04	(0.79)	0.14	(0.30)	0.11	(0.45)	-0.01	(0.97)	0.03	(0.81)	-0.25	(0.06)	-0.22	(0.10)
BCS	0.14	(0.34)	0.16	(0.27)	0.12	(0.42)	0.00	(0.98)	-0.06	(0.70)	-0.13	(0.36)	-0.28	(0.05)	-0.29	(0.04)
Delta body weight	-0.09	(0.49)	-0.27	(0.06)	-0.01	(0.95)	0.07	(0.64)	0.09	(0.53)	0.04	(0.78)	-0.04	(0.75)	-0.07	(0.62)
Delta BCS	0.18	(0.23)	-0.04	(0.81)	0.12	(0.42)	0.00	(0.98)	-0.29	(0.05)	-0.03	(0.83)	0.07	(0.61)	-0.08	(0.61)
Lactation week 4																
Dry matter intake [kg DM]	-0.12	(0.40)	-0.11	(0.44)	-0.02	(0.91)	0.13	(0.35)	0.07	(0.61)	0.11	(0.44)	0.22	(0.12)	0.14	(0.34)
Energy intake [MJ NE_L]	-0.10	(0.50)	-0.08	(0.60)	0.04	(0.81)	0.14	(0.34)	-0.01	(0.95)	0.13	(0.36)	0.27	(0.05)	0.18	(0.20)
Dry matter intake per metabolic BW [kg DM/ $BW^{0.75}$]	-0.13	(0.37)	-0.10	(0.49)	0.00	(0.99)	0.07	(0.65)	0.02	(0.89)	0.15	(0.31)	0.37	(0.01)	0.24	(0.09)
Energy intake per metabolic BW [MJ NE_L/ $BW^{0.75}$]	-0.11	(0.46)	-0.08	(0.58)	0.04	(0.81)	0.07	(0.63)	-0.02	(0.89)	0.16	(0.28)	0.37	(0.01)	0.25	(0.07)
Energy balance [MJ NE_L]	-0.19	(0.20)	-0.17	(0.26)	-0.24	(0.09)	0.25	(0.09)	0.04	(0.77)	0.26	(0.07)	0.45	(0.00)	0.39	(0.01)
Maintenance [MJ NE_L]	-0.04	(0.76)	-0.07	(0.62)	0.03	(0.86)	0.10	(0.50)	0.00	(0.98)	-0.01	(0.92)	-0.07	(0.62)	-0.10	(0.46)
Milk [MJ NE_L]	0.19	(0.19)	0.12	(0.42)	0.24	(0.09)	-0.07	(0.65)	-0.09	(0.53)	-0.14	(0.31)	-0.24	(0.09)	-0.31	(0.03)
BCS	0.08	(0.59)	0.06	(0.68)	0.07	(0.65)	0.15	(0.32)	-0.03	(0.82)	-0.09	(0.55)	-0.25	(0.07)	-0.30	(0.03)
Delta body weight	0.18	(0.20)	0.24	(0.09)	0.00	(0.99)	-0.21	(0.15)	-0.09	(0.54)	-0.02	(0.88)	0.10	(0.48)	0.15	(0.28)
Delta BCS	-0.05	(0.76)	-0.26	(0.08)	-0.02	(0.89)	0.28	(0.06)	-0.12	(0.43)	0.03	(0.85)	0.05	(0.73)	-0.01	(0.97)
Lactation week 5																
Dry matter intake [kg DM]	-0.21	(0.12)	-0.12	(0.38)	-0.12	(0.38)	0.24	(0.07)	0.12	(0.37)	0.22	(0.09)	0.23	(0.08)	0.29	(0.02)
Energy intake [MJ NE_L]	-0.23	(0.08)	-0.12	(0.38)	-0.12	(0.38)	0.25	(0.06)	0.12	(0.37)	0.21	(0.10)	0.19	(0.15)	0.23	(0.07)
Dry matter intake per metabolic BW [kg DM/ $BW^{0.75}$]	-0.33	(0.01)	-0.18	(0.17)	-0.23	(0.09)	0.17	(0.20)	0.22	(0.11)	0.29	(0.03)	0.27	(0.04)	0.33	(0.01)
Energy intake per metabolic BW [MJ NE_L/ $BW^{0.75}$]	-0.33	(0.01)	-0.17	(0.21)	-0.23	(0.10)	0.20	(0.13)	0.19	(0.15)	0.31	(0.02)	0.26	(0.05)	0.32	(0.01)
Energy balance [MJ NE_L]	-0.06	(0.66)	-0.05	(0.71)	-0.11	(0.43)	-0.06	(0.67)	-0.12	(0.37)	0.19	(0.16)	0.32	(0.01)	0.39	(0.00)
Maintenance [MJ NE_L]	-0.01	(0.92)	-0.05	(0.71)	0.09	(0.53)	0.12	(0.39)	-0.09	(0.52)	0.01	(0.96)	0.11	(0.42)	0.00	(0.99)
Milk [MJ NE_L]	0.06	(0.67)	-0.02	(0.90)	0.11	(0.40)	0.16	(0.25)	0.09	(0.51)	-0.07	(0.61)	-0.27	(0.04)	-0.25	(0.06)
BCS	0.13	(0.32)	0.10	(0.48)	0.08	(0.56)	0.02	(0.91)	-0.02	(0.88)	-0.11	(0.40)	-0.10	(0.45)	-0.19	(0.15)
Delta body weight	-0.03	(0.85)	0.05	(0.71)	0.02	(0.86)	-0.06	(0.67)	-0.20	(0.14)	-0.05	(0.72)	0.24	(0.07)	0.22	(0.10)

Delta BCS	0.05	(0.71)	-0.17	(0.22)	-0.01	(0.96)	0.02	(0.87)	-0.05	(0.71)	0.06	(0.67)	0.17	(0.20)	0.06	(0.68)
Lactation week 6																
Dry matter intake [kg DM]	-0.05	(0.70)	-0.10	(0.47)	-0.14	(0.30)	0.17	(0.22)	0.04	(0.79)	0.09	(0.49)	0.30	(0.02)	0.27	(0.04)
Energy intake [MJ NE_L]	-0.09	(0.50)	-0.07	(0.60)	-0.14	(0.29)	0.24	(0.08)	0.01	(0.94)	0.11	(0.41)	0.25	(0.06)	0.24	(0.08)
Dry matter intake per metabolic BW [kg DM/ $BW^{0.75}$]	-0.09	(0.50)	-0.17	(0.22)	-0.14	(0.28)	0.12	(0.37)	0.07	(0.63)	0.07	(0.62)	0.13	(0.34)	0.05	(0.72)
Energy intake per metabolic BW [MJ NE_L/ $BW^{0.75}$]	-0.19	(0.16)	-0.17	(0.21)	-0.21	(0.12)	0.18	(0.19)	0.12	(0.39)	0.18	(0.17)	0.18	(0.17)	0.20	(0.15)
Energy balance [MJ NE_L]	0.00	(0.98)	0.01	(0.92)	-0.04	(0.75)	0.16	(0.24)	0.02	(0.86)	0.01	(0.92)	0.18	(0.19)	0.19	(0.17)
Maintenance [MJ NE_L]	0.07	(0.61)	0.03	(0.84)	0.07	(0.60)	0.05	(0.73)	-0.08	(0.54)	-0.05	(0.69)	0.03	(0.82)	0.03	(0.81)
Milk [MJ NE_L]	-0.02	(0.86)	-0.04	(0.79)	-0.05	(0.70)	0.04	(0.78)	0.07	(0.61)	0.05	(0.73)	-0.04	(0.76)	-0.10	(0.44)
BCS	0.14	(0.30)	0.14	(0.31)	0.09	(0.48)	0.04	(0.78)	0.00	(0.97)	-0.20	(0.13)	-0.16	(0.24)	-0.24	(0.07)
Delta body weight	0.00	(0.99)	0.02	(0.91)	0.03	(0.82)	0.04	(0.75)	-0.11	(0.44)	-0.03	(0.80)	-0.08	(0.57)	-0.05	(0.70)
Delta BCS	-0.03	(0.82)	-0.08	(0.54)	0.00	(0.98)	0.10	(0.45)	-0.07	(0.61)	0.06	(0.63)	0.10	(0.46)	0.20	(0.13)

Suppl. Table S5: Pearson correlation coefficients (r) and *p*-values (in brackets) between fatty acids (% of total fatty acids) of hair in week 4 and 8 and parameters of energy availability in week 2 to 6 on farm 2 (SIM)

	Hair sample week 4															
Lactation week 2	C10:0		C12:0		C14:0		C16:0		C18:0		C18:1*cis*-9		C18:2*n*-6		C18:3*n*-3	
Dry matter intake [kg DM]	0.31	(0.07)	0.31	(0.07)	0.33	(0.05)	-0.46	(0.00)	-0.29	(0.08)	-0.10	(0.55)	0.18	(0.30)	0.21	(0.22)
Energy intake [MJ NE_L]	0.27	(0.10)	0.28	(0.10)	0.30	(0.07)	-0.43	(0.01)	-0.27	(0.11)	-0.07	(0.69)	0.23	(0.17)	0.26	(0.12)
Dry matter intake per metabolic BW [kg DM/ $BW^{0.75}$]	0.22	(0.20)	0.21	(0.22)	0.21	(0.22)	-0.37	(0.03)	-0.16	(0.35)	-0.04	(0.83)	0.28	(0.10)	0.30	(0.08)
Energy intake per metabolic BW [MJ NE_L/ $BW^{0.75}$]	0.16	(0.33)	0.20	(0.24)	0.18	(0.28)	-0.36	(0.03)	-0.17	(0.33)	-0.01	(0.95)	0.34	(0.04)	0.33	(0.05)
Energy balance [MJ NE_L]	0.21	(0.23)	0.11	(0.54)	0.10	(0.55)	-0.13	(0.44)	-0.06	(0.72)	0.03	(0.85)	-0.06	(0.71)	-0.11	(0.51)
Maintenance [MJ NE_L]	0.18	(0.31)	0.13	(0.47)	0.21	(0.22)	-0.12	(0.48)	-0.29	(0.10)	-0.07	(0.70)	-0.13	(0.46)	-0.12	(0.51)
Milk [MJ NE_L]	0.22	(0.21)	0.33	(0.05)	0.27	(0.11)	-0.33	(0.05)	-0.35	(0.04)	-0.16	(0.35)	0.35	(0.03)	0.34	(0.04)
BCS	-0.20	(0.27)	-0.18	(0.33)	-0.07	(0.70)	0.25	(0.18)	0.14	(0.46)	-0.08	(0.67)	-0.36	(0.04)	-0.35	(0.05)
Delta body weight	-0.03	(0.89)	-0.06	(0.74)	-0.06	(0.77)	0.13	(0.49)	0.18	(0.36)	0.01	(0.96)	-0.25	(0.19)	-0.32	(0.09)
Delta BCS	0.14	(0.47)	0.13	(0.49)	0.20	(0.30)	-0.15	(0.44)	-0.15	(0.44)	-0.15	(0.43)	0.05	(0.80)	-0.11	(0.59)

	Hair sample week 8															
Lactation week 2	C10:0		C12:0		C14:0		C16:0		C18:0		C18:1*cis*-9		C18:2*n*-6		C18:3*n*-3	
Dry matter intake [kg DM]	-0.07	(0.67)	0.12	(0.46)	0.12	(0.45)	0.00	(0.98)	0.13	(0.43)	-0.25	(0.12)	0.26	(0.10)	0.45	(0.00)
Energy intake [MJ NE_L]	-0.05	(0.78)	0.16	(0.33)	0.17	(0.29)	-0.03	(0.84)	0.11	(0.49)	-0.31	(0.05)	0.27	(0.09)	0.46	(0.00)
Dry matter intake per metabolic BW [kg DM/ $BW^{0.75}$]	-0.07	(0.68)	0.15	(0.35)	0.15	(0.36)	-0.06	(0.72)	0.19	(0.25)	-0.37	(0.02)	0.26	(0.10)	0.41	(0.01)
Energy intake per metabolic BW [MJ NE_L/ $BW^{0.75}$]	-0.06	(0.71)	0.20	(0.20)	0.16	(0.30)	-0.07	(0.66)	0.16	(0.32)	-0.37	(0.02)	0.29	(0.06)	0.45	(0.00)
Energy balance [MJ NE_L]	-0.01	(0.93)	0.13	(0.45)	0.01	(0.94)	0.18	(0.27)	0.17	(0.30)	-0.16	(0.32)	-0.17	(0.31)	-0.06	(0.73)
Maintenance [MJ NE_L]	0.17	(0.30)	-0.07	(0.70)	0.04	(0.80)	0.11	(0.49)	-0.12	(0.47)	0.12	(0.46)	-0.23	(0.17)	-0.12	(0.45)
Milk [MJ NE_L]	0.05	(0.74)	0.18	(0.27)	0.17	(0.29)	-0.26	(0.11)	-0.17	(0.28)	-0.25	(0.11)	0.36	(0.02)	0.30	(0.06)
BCS	0.00	(1.00)	-0.14	(0.42)	0.03	(0.85)	-0.11	(0.54)	-0.12	(0.50)	0.16	(0.34)	-0.14	(0.39)	-0.21	(0.21)
Delta body weight	-0.21	(0.27)	0.02	(0.94)	-0.04	(0.84)	0.17	(0.35)	0.09	(0.61)	-0.07	(0.72)	-0.18	(0.32)	-0.33	(0.06)
Delta BCS	0.03	(0.85)	0.14	(0.46)	0.10	(0.58)	0.06	(0.74)	0.10	(0.58)	-0.35	(0.05)	-0.14	(0.46)	-0.08	(0.68)

Lactation week 3

Dry matter intake [kg DM]	-0.03	(0.86)	-0.11	(0.51)	-0.13	(0.43)	-0.03	(0.86)	0.10	(0.52)	-0.01	(0.95)	0.37	(0.02)	0.46	(0.00)
Energy intake [MJ NE_L]	-0.04	(0.82)	-0.12	(0.46)	-0.11	(0.50)	-0.02	(0.92)	0.08	(0.61)	-0.01	(0.96)	0.36	(0.02)	0.43	(0.00)
Dry matter intake per metabolic BW [kg DM/ $BW^{0.75}$]	-0.01	(0.94)	-0.10	(0.55)	-0.07	(0.66)	-0.09	(0.59)	0.18	(0.26)	-0.12	(0.44)	0.37	(0.02)	0.45	(0.00)
Energy intake per metabolic BW [MJ NE_L/ $BW^{0.75}$]	-0.04	(0.82)	-0.12	(0.44)	-0.07	(0.67)	-0.04	(0.82)	0.16	(0.30)	-0.10	(0.52)	0.39	(0.01)	0.45	(0.00)
Energy balance [MJ NE_L]	-0.30	(0.06)	-0.27	(0.09)	-0.22	(0.18)	0.16	(0.32)	0.39	(0.01)	0.11	(0.52)	0.03	(0.87)	0.08	(0.62)
Maintenance [MJ NE_L]	0.08	(0.62)	0.12	(0.47)	-0.09	(0.57)	0.03	(0.83)	-0.16	(0.33)	0.30	(0.06)	-0.21	(0.19)	-0.13	(0.40)
Milk [MJ NE_L]	0.34	(0.03)	0.29	(0.07)	0.23	(0.14)	-0.34	(0.03)	-0.32	(0.04)	-0.36	(0.02)	0.20	(0.21)	0.22	(0.17)
BCS	-0.02	(0.92)	-0.07	(0.70)	0.07	(0.68)	-0.14	(0.42)	-0.11	(0.50)	0.06	(0.74)	-0.05	(0.78)	-0.14	(0.40)
Delta body weight	0.10	(0.53)	0.20	(0.22)	0.25	(0.12)	-0.15	(0.35)	-0.02	(0.90)	-0.39	(0.01)	0.08	(0.63)	0.10	(0.53)
Delta BCS	0.29	(0.09)	0.35	(0.05)	0.22	(0.21)	-0.17	(0.34)	-0.18	(0.30)	-0.09	(0.59)	-0.06	(0.75)	-0.14	(0.41)
Lactation week 4																
Dry matter intake [kg DM]	-0.06	(0.66)	0.03	(0.84)	0.02	(0.90)	-0.25	(0.09)	0.01	(0.97)	-0.20	(0.17)	0.28	(0.05)	0.41	(0.00)
Energy intake [MJ NE_L]	-0.10	(0.50)	-0.02	(0.90)	0.00	(0.99)	-0.20	(0.17)	0.01	(0.94)	-0.15	(0.30)	0.28	(0.05)	0.39	(0.00)
Dry matter intake per metabolic BW [kg DM/ $BW^{0.75}$]	-0.14	(0.33)	0.01	(0.95)	-0.02	(0.88)	-0.06	(0.70)	0.17	(0.23)	-0.27	(0.05)	0.31	(0.03)	0.37	(0.01)
Energy intake per metabolic BW [MJ NE_L/ $BW^{0.75}$]	-0.15	(0.30)	0.04	(0.81)	0.00	(0.98)	-0.06	(0.66)	0.18	(0.19)	-0.27	(0.05)	0.33	(0.02)	0.39	(0.00)
Energy balance [MJ NE_L]	-0.27	(0.06)	-0.04	(0.77)	-0.12	(0.40)	0.29	(0.04)	0.26	(0.06)	0.04	(0.80)	0.10	(0.50)	0.09	(0.54)
Maintenance [MJ NE_L]	0.09	(0.54)	-0.09	(0.55)	-0.05	(0.75)	-0.04	(0.80)	-0.18	(0.22)	0.21	(0.16)	-0.05	(0.75)	0.03	(0.82)
Milk [MJ NE_L]	0.21	(0.15)	0.11	(0.46)	0.17	(0.25)	-0.39	(0.00)	-0.19	(0.19)	-0.22	(0.12)	0.16	(0.27)	0.23	(0.10)
BCS	-0.08	(0.61)	-0.26	(0.08)	-0.16	(0.27)	0.05	(0.75)	0.07	(0.63)	0.16	(0.29)	-0.08	(0.58)	-0.14	(0.33)
Delta body weight	0.28	(0.05)	0.24	(0.10)	0.27	(0.06)	-0.22	(0.13)	-0.08	(0.59)	-0.24	(0.09)	-0.15	(0.31)	-0.06	(0.66)
Delta BCS	0.03	(0.83)	0.12	(0.42)	0.00	(0.98)	-0.06	(0.72)	-0.02	(0.88)	-0.11	(0.45)	0.03	(0.86)	0.06	(0.70)
Lactation week 5																
Dry matter intake [kg DM]	0.04	(0.78)	0.08	(0.60)	0.13	(0.37)	-0.15	(0.29)	-0.04	(0.78)	-0.27	(0.05)	0.36	(0.01)	0.43	(0.00)
Energy intake [MJ NE_L]	0.06	(0.69)	0.03	(0.85)	0.11	(0.46)	-0.11	(0.43)	-0.05	(0.73)	-0.16	(0.27)	0.37	(0.01)	0.45	(0.00)
Dry matter intake per metabolic BW [kg DM/ $BW^{0.75}$]	0.06	(0.66)	0.17	(0.24)	0.14	(0.34)	-0.13	(0.37)	0.07	(0.63)	-0.33	(0.02)	0.33	(0.02)	0.39	(0.00)
Energy intake per metabolic BW [MJ NE_L/ $BW^{0.75}$]	0.07	(0.64)	0.14	(0.32)	0.11	(0.45)	-0.11	(0.44)	0.07	(0.61)	-0.30	(0.03)	0.31	(0.02)	0.38	(0.00)
Energy balance [MJ NE_L]	-0.08	(0.57)	0.25	(0.08)	0.15	(0.30)	0.12	(0.40)	-0.07	(0.63)	-0.17	(0.22)	0.07	(0.61)	0.09	(0.52)
Maintenance [MJ NE_L]	0.13	(0.35)	-0.05	(0.71)	0.05	(0.74)	-0.04	(0.76)	-0.22	(0.13)	0.19	(0.17)	0.03	(0.83)	0.12	(0.39)
Milk [MJ NE_L]	0.18	(0.20)	0.07	(0.63)	0.16	(0.26)	-0.38	(0.00)	-0.09	(0.50)	-0.19	(0.17)	0.34	(0.01)	0.39	(0.00)
BCS	-0.15	(0.28)	-0.25	(0.07)	-0.20	(0.16)	0.20	(0.15)	0.09	(0.50)	0.16	(0.25)	-0.10	(0.45)	-0.17	(0.22)

Delta body weight	0.24	(0.09)	0.22	(0.12)	0.15	(0.30)	0.08	(0.57)	-0.12	(0.41)	-0.08	(0.57)	0.08	(0.57)	0.10	(0.45)
Delta BCS	-0.09	(0.55)	0.07	(0.63)	0.04	(0.81)	0.17	(0.24)	0.01	(0.97)	-0.03	(0.86)	-0.06	(0.67)	-0.05	(0.71)
Lactation week 6																
Dry matter intake [kg DM]	-0.04	(0.80)	-0.07	(0.63)	0.08	(0.58)	-0.18	(0.19)	-0.02	(0.91)	-0.14	(0.32)	0.42	(0.00)	0.47	(0.00)
Energy intake [MJ NE_L]	0.00	(1.00)	-0.06	(0.70)	0.08	(0.56)	-0.20	(0.14)	-0.03	(0.82)	-0.13	(0.34)	0.41	(0.00)	0.47	(0.00)
Dry matter intake per metabolic BW [kg DM/ $BW^{0.75}$]	-0.04	(0.75)	-0.02	(0.90)	0.03	(0.83)	-0.11	(0.41)	0.09	(0.49)	-0.20	(0.15)	0.34	(0.01)	0.36	(0.01)
Energy intake per metabolic BW [MJ NE_L/ $BW^{0.75}$]	0.00	(1.00)	0.00	(0.99)	0.04	(0.80)	-0.14	(0.31)	0.08	(0.58)	-0.19	(0.16)	0.33	(0.01)	0.35	(0.01)
Energy balance [MJ NE_L]	0.00	(0.99)	0.17	(0.22)	0.07	(0.61)	0.11	(0.45)	-0.01	(0.92)	0.01	(0.94)	0.00	(1.00)	-0.02	(0.88)
Maintenance [MJ NE_L]	0.15	(0.28)	-0.06	(0.69)	0.05	(0.74)	-0.02	(0.86)	-0.20	(0.16)	0.20	(0.15)	0.02	(0.87)	0.12	(0.40)
Milk [MJ NE_L]	0.12	(0.38)	0.06	(0.69)	0.14	(0.30)	-0.35	(0.01)	-0.12	(0.38)	-0.21	(0.13)	0.36	(0.01)	0.39	(0.00)
BCS	-0.18	(0.21)	-0.23	(0.11)	-0.18	(0.19)	0.13	(0.35)	0.02	(0.88)	0.21	(0.12)	-0.10	(0.47)	-0.22	(0.12)
Delta body weight	0.09	(0.54)	0.04	(0.75)	-0.01	(0.95)	0.07	(0.60)	0.26	(0.06)	-0.01	(0.96)	-0.20	(0.15)	-0.15	(0.27)
Delta BCS	0.01	(0.94)	0.11	(0.44)	0.13	(0.35)	-0.06	(0.65)	-0.21	(0.12)	0.13	(0.33)	0.00	(0.99)	-0.05	(0.71)

Suppl. Table S6: Pearson correlation coefficients (r) and *p*-values (in brackets) between fatty acids (% of total fatty acids) of hair in week 4 and 8 and parameters of energy availability in week 2 to 6 on farm 3 (HOL)

	Hair sample week 4															
Lactation week 2	C10:0		C12:0		C14:0		C16:0		C18:0		C18:1*cis*-9		C18:2*n*-6		C18:3*n*-3	
Dry matter intake [kg DM]	-0.36	(0.03)	-0.03	(0.88)	0.09	(0.60)	0.23	(0.17)	0.32	(0.06)	0.08	(0.66)	0.13	(0.44)	0.12	(0.50)
Energy intake [MJ NE_L]	-0.36	(0.03)	-0.01	(0.94)	0.10	(0.56)	0.22	(0.19)	0.31	(0.07)	0.07	(0.69)	0.15	(0.39)	0.12	(0.48)
Dry matter intake per metabolic BW [kg DM/ $BW^{0.75}$]	-0.30	(0.07)	-0.03	(0.88)	0.03	(0.86)	0.20	(0.24)	0.32	(0.06)	0.11	(0.53)	0.30	(0.08)	0.24	(0.17)
Energy intake per metabolic BW [MJ NE_L/ $BW^{0.75}$]	-0.30	(0.08)	-0.01	(0.96)	0.03	(0.88)	0.19	(0.28)	0.36	(0.04)	0.08	(0.64)	0.28	(0.10)	0.21	(0.23)
Energy balance [MJ NE_L]	-0.30	(0.07)	0.10	(0.56)	0.15	(0.38)	0.09	(0.62)	0.15	(0.39)	-0.01	(0.97)	0.21	(0.23)	0.21	(0.22)
Maintenance [MJ NE_L]	-0.17	(0.34)	-0.18	(0.30)	-0.06	(0.73)	0.33	(0.06)	0.18	(0.31)	0.16	(0.37)	-0.23	(0.19)	-0.22	(0.21)
Milk [MJ NE_L]	-0.01	(0.94)	0.04	(0.83)	0.01	(0.96)	0.00	(1.00)	0.02	(0.91)	-0.03	(0.87)	0.10	(0.55)	-0.01	(0.95)
BCS	-0.06	(0.74)	-0.14	(0.41)	-0.05	(0.78)	0.18	(0.30)	0.10	(0.57)	0.11	(0.51)	-0.26	(0.12)	-0.31	(0.07)
Delta body weight	-0.06	(0.74)	-0.18	(0.32)	-0.12	(0.49)	0.09	(0.62)	0.27	(0.13)	0.17	(0.35)	0.13	(0.46)	0.10	(0.56)
Delta BCS	-0.09	(0.59)	-0.16	(0.37)	-0.13	(0.47)	0.28	(0.10)	0.21	(0.22)	0.15	(0.40)	-0.22	(0.20)	-0.19	(0.28)

	Hair sample week 8															
Lactation week 2	C10:0		C12:0		C14:0		C16:0		C18:0		C18:1*cis*-9		C18:2*n*-6		C18:3*n*-3	
Dry matter intake [kg DM]	-0.03	(0.83)	-0.20	(0.15)	-0.18	(0.20)	0.26	(0.07)	0.23	(0.10)	0.13	(0.34)	-0.08	(0.55)	-0.03	(0.84)
Energy intake [MJ NE_L]	-0.04	(0.80)	-0.18	(0.20)	-0.12	(0.39)	0.24	(0.10)	0.19	(0.18)	0.10	(0.48)	-0.10	(0.48)	-0.08	(0.58)
Dry matter intake per metabolic BW [kg DM/ $BW^{0.75}$]	0.04	(0.77)	-0.09	(0.55)	-0.10	(0.46)	0.14	(0.34)	0.00	(1.00)	0.04	(0.77)	0.06	(0.69)	0.01	(0.95)
Energy intake per metabolic BW [MJ NE_L/ $BW^{0.75}$]	0.06	(0.68)	-0.05	(0.74)	-0.07	(0.60)	0.11	(0.47)	-0.03	(0.86)	0.01	(0.95)	0.05	(0.73)	-0.01	(0.93)
Energy balance [MJ NE_L]	-0.04	(0.78)	-0.01	(0.92)	-0.08	(0.57)	0.03	(0.82)	0.03	(0.86)	-0.12	(0.41)	0.00	(0.99)	0.04	(0.76)
Maintenance [MJ NE_L]	-0.15	(0.31)	-0.34	(0.01)	-0.24	(0.10)	0.21	(0.15)	0.36	(0.01)	0.27	(0.06)	-0.28	(0.04)	-0.16	(0.28)
Milk [MJ NE_L]	-0.01	(0.93)	-0.04	(0.79)	0.02	(0.86)	0.10	(0.50)	0.15	(0.29)	0.11	(0.42)	0.04	(0.77)	0.03	(0.83)
BCS	-0.04	(0.76)	-0.30	(0.03)	-0.28	(0.05)	0.08	(0.58)	0.28	(0.04)	0.23	(0.10)	-0.17	(0.21)	-0.11	(0.45)
Delta body weight	-0.01	(0.96)	-0.16	(0.28)	-0.13	(0.36)	0.00	(1.00)	0.10	(0.51)	0.10	(0.51)	-0.03	(0.84)	0.02	(0.91)
Delta BCS	0.00	(0.97)	-0.20	(0.16)	-0.20	(0.16)	0.20	(0.16)	0.31	(0.03)	0.18	(0.20)	-0.15	(0.28)	-0.12	(0.38)
Lactation week 3																

Dry matter intake [kg DM]	0.02	(0.92)	-0.16	(0.28)	-0.06	(0.68)	0.26	(0.08)	0.05	(0.74)	0.09	(0.53)	-0.10	(0.51)	-0.09	(0.54)
Energy intake [MJ NE_L]	0.07	(0.61)	-0.03	(0.85)	0.05	(0.74)	0.16	(0.28)	0.02	(0.88)	0.00	(0.98)	-0.11	(0.45)	-0.15	(0.32)
Dry matter intake per metabolic BW [kg DM/ $BW^{0.75}$]	0.05	(0.75)	0.12	(0.42)	0.11	(0.47)	0.08	(0.57)	-0.03	(0.85)	-0.17	(0.24)	0.08	(0.57)	0.03	(0.82)
Energy intake per metabolic BW [MJ NE_L/ $BW^{0.75}$]	0.13	(0.38)	0.15	(0.31)	0.10	(0.49)	0.01	(0.94)	-0.16	(0.28)	-0.13	(0.37)	0.08	(0.60)	0.01	(0.92)
Energy balance [MJ NE_L]	0.03	(0.85)	0.12	(0.41)	0.07	(0.65)	-0.08	(0.60)	-0.14	(0.34)	-0.18	(0.22)	0.16	(0.25)	0.12	(0.43)
Maintenance [MJ NE_L]	-0.05	(0.75)	-0.40	(0.00)	-0.31	(0.03)	0.20	(0.18)	0.36	(0.01)	0.37	(0.01)	-0.23	(0.10)	-0.09	(0.51)
Milk [MJ NE_L]	-0.01	(0.93)	-0.10	(0.50)	-0.04	(0.79)	0.22	(0.13)	0.20	(0.15)	0.17	(0.23)	-0.19	(0.18)	-0.14	(0.32)
BCS	-0.08	(0.57)	-0.32	(0.02)	-0.28	(0.05)	0.12	(0.41)	0.30	(0.03)	0.24	(0.08)	-0.22	(0.12)	-0.16	(0.27)
Delta body weight	0.16	(0.27)	0.11	(0.44)	0.10	(0.49)	-0.25	(0.08)	-0.12	(0.41)	-0.04	(0.78)	0.46	(0.00)	0.21	(0.14)
Delta BCS	-0.10	(0.48)	0.09	(0.55)	0.15	(0.28)	-0.04	(0.81)	-0.18	(0.21)	-0.06	(0.69)	0.03	(0.82)	-0.10	(0.49)
Lactation week 4																
Dry matter intake [kg DM]	0.16	(0.26)	0.00	(1.00)	-0.02	(0.92)	0.00	(1.00)	0.01	(0.96)	0.02	(0.91)	0.12	(0.41)	0.03	(0.81)
Energy intake [MJ NE_L]	0.16	(0.27)	0.00	(1.00)	-0.01	(0.94)	0.00	(1.00)	0.01	(0.92)	0.02	(0.92)	0.12	(0.42)	0.03	(0.83)
Dry matter intake per metabolic BW [kg DM/ $BW^{0.75}$]	0.26	(0.07)	0.21	(0.15)	0.11	(0.46)	-0.15	(0.29)	-0.26	(0.06)	-0.16	(0.26)	0.26	(0.07)	0.14	(0.35)
Energy intake per metabolic BW [MJ NE_L/ $BW^{0.75}$]	0.25	(0.08)	0.21	(0.14)	0.11	(0.43)	-0.15	(0.31)	-0.26	(0.06)	-0.17	(0.23)	0.27	(0.06)	0.15	(0.30)
Energy balance [MJ NE_L]	0.18	(0.21)	0.22	(0.12)	0.06	(0.69)	-0.22	(0.13)	-0.32	(0.02)	-0.25	(0.08)	0.17	(0.23)	0.04	(0.78)
Maintenance [MJ NE_L]	-0.13	(0.36)	-0.42	(0.00)	-0.30	(0.04)	0.20	(0.16)	0.38	(0.01)	0.39	(0.01)	-0.21	(0.15)	-0.09	(0.55)
Milk [MJ NE_L]	0.07	(0.64)	-0.15	(0.31)	-0.02	(0.91)	0.13	(0.37)	0.22	(0.14)	0.22	(0.14)	-0.09	(0.53)	-0.09	(0.55)
BCS	-0.11	(0.43)	-0.31	(0.03)	-0.26	(0.07)	0.14	(0.34)	0.33	(0.02)	0.24	(0.09)	-0.24	(0.10)	-0.17	(0.23)
Delta body weight	-0.18	(0.22)	0.09	(0.52)	0.23	(0.11)	-0.14	(0.35)	0.01	(0.95)	-0.06	(0.66)	0.20	(0.16)	0.06	(0.70)
Delta BCS	-0.09	(0.54)	0.08	(0.60)	0.14	(0.33)	-0.03	(0.82)	-0.21	(0.14)	-0.04	(0.80)	0.04	(0.79)	-0.02	(0.87)
Lactation week 5																
Dry matter intake [kg DM]	0.23	(0.10)	-0.10	(0.50)	-0.05	(0.72)	0.01	(0.95)	0.12	(0.42)	0.04	(0.79)	0.06	(0.68)	0.00	(0.97)
Energy intake [MJ NE_L]	0.25	(0.08)	-0.13	(0.37)	-0.09	(0.54)	0.01	(0.93)	0.01	(0.92)	0.10	(0.47)	0.08	(0.58)	0.03	(0.84)
Dry matter intake per metabolic BW [kg DM/ $BW^{0.75}$]	0.34	(0.02)	0.13	(0.36)	0.10	(0.51)	-0.16	(0.28)	-0.16	(0.26)	-0.18	(0.22)	0.18	(0.20)	0.07	(0.61)
Energy intake per metabolic BW [MJ NE_L/ $BW^{0.75}$]	0.37	(0.01)	0.10	(0.50)	0.05	(0.75)	-0.16	(0.26)	-0.22	(0.13)	-0.10	(0.49)	0.21	(0.13)	0.09	(0.54)
Energy balance [MJ NE_L]	0.03	(0.82)	0.05	(0.71)	0.04	(0.77)	-0.13	(0.37)	-0.03	(0.81)	-0.18	(0.21)	0.04	(0.76)	-0.01	(0.94)
Maintenance [MJ NE_L]	-0.15	(0.28)	-0.43	(0.00)	-0.31	(0.03)	0.23	(0.11)	0.32	(0.02)	0.42	(0.00)	-0.23	(0.10)	-0.10	(0.48)
Milk [MJ NE_L]	0.20	(0.16)	-0.06	(0.68)	-0.02	(0.86)	0.07	(0.64)	0.12	(0.41)	0.15	(0.31)	0.04	(0.80)	0.01	(0.96)
BCS	-0.16	(0.28)	-0.29	(0.04)	-0.24	(0.09)	0.12	(0.43)	0.27	(0.06)	0.25	(0.09)	-0.12	(0.41)	-0.08	(0.59)

Delta body weight	0.09	(0.53)	0.06	(0.69)	0.09	(0.51)	0.01	(0.94)	-0.07	(0.65)	-0.13	(0.37)	0.00	(0.99)	0.02	(0.87)
Delta BCS	0.13	(0.38)	0.15	(0.31)	0.13	(0.39)	-0.12	(0.43)	0.00	(0.98)	-0.06	(0.68)	0.07	(0.61)	0.05	(0.75)
Lactation week 6																
Dry matter intake [kg DM]	0.04	(0.79)	-0.04	(0.75)	0.03	(0.82)	0.00	(0.98)	-0.03	(0.82)	0.09	(0.51)	0.19	(0.17)	0.18	(0.20)
Energy intake [MJ NE_L]	0.10	(0.46)	-0.11	(0.42)	-0.02	(0.88)	0.02	(0.87)	0.03	(0.81)	0.11	(0.43)	0.15	(0.30)	0.12	(0.40)
Dry matter intake per metabolic BW [kg DM/ $BW^{0.75}$]	0.27	(0.06)	0.04	(0.81)	0.04	(0.75)	-0.14	(0.32)	-0.15	(0.30)	-0.08	(0.60)	0.28	(0.04)	0.19	(0.17)
Energy intake per metabolic BW [MJ NE_L/ $BW^{0.75}$]	0.28	(0.04)	0.05	(0.72)	0.05	(0.71)	-0.17	(0.24)	-0.16	(0.25)	-0.07	(0.60)	0.27	(0.05)	0.18	(0.19)
Energy balance [MJ NE_L]	0.16	(0.27)	-0.02	(0.89)	0.06	(0.68)	-0.23	(0.11)	-0.07	(0.62)	-0.15	(0.30)	0.02	(0.91)	-0.01	(0.96)
Maintenance [MJ NE_L]	-0.14	(0.32)	-0.44	(0.00)	-0.32	(0.02)	0.23	(0.10)	0.37	(0.01)	0.41	(0.00)	-0.23	(0.09)	-0.10	(0.46)
Milk [MJ NE_L]	0.00	(0.99)	-0.01	(0.92)	-0.02	(0.87)	0.11	(0.42)	0.09	(0.53)	0.16	(0.26)	0.17	(0.22)	0.13	(0.37)
BCS	-0.07	(0.61)	-0.31	(0.03)	-0.26	(0.06)	0.09	(0.55)	0.29	(0.04)	0.22	(0.12)	-0.16	(0.26)	-0.13	(0.38)
Delta body weight	0.10	(0.47)	-0.02	(0.87)	-0.10	(0.49)	0.02	(0.91)	0.07	(0.62)	0.02	(0.89)	0.02	(0.86)	-0.03	(0.82)
Delta BCS	0.22	(0.12)	-0.24	(0.09)	-0.18	(0.21)	-0.01	(0.92)	0.09	(0.52)	0.00	(0.99)	-0.20	(0.15)	-0.20	(0.16)
